# Supplementary material for: Overweight or obesity in children born after assisted reproductive technologies in Denmark: A population-based cohort study
Source: PLoS Med. 2023 Dec 19;20(12):e1004324. doi: 10.1371/journal.pmed.1004324 (PMC10729995; doi:10.1371/journal.pmed.1004324)
Supplement: S1 STROBE Checklist — (PDF) [file pmed.1004324.s001.pdf]

**S1 STROBE Checklist**

# STROBE Statement—Checklist of items that should be included in reports of ***cohort studies***

| | Item No | Recommendation | Page No |
|---|---|---|---|
| **Title and abstract** | 1 | (*a*) Indicate the study's design with a commonly used term in the title or the abstract | Title |
| | | (*b*) Provide in the abstract an informative and balanced summary of what was done and what was found | Abstract |
| **Introduction** | | | |
| Background/rationale | 2 | Explain the scientific background and rationale for the investigation being reported | Introduction, paragraph 1-2 |
| Objectives | 3 | State specific objectives, including any prespecified hypotheses | Introduction, paragraph 3 |
| **Methods** | | | |
| Study design | 4 | Present key elements of study design early in the paper | Methods, setting and data sources, paragraph 1 |
| Setting | 5 | Describe the setting, locations, and relevant dates, including periods of recruitment, exposure, follow-up, and data collection | Methods, setting and data sources, paragraph 1 study cohorts, paragraph 1, overweight, obesity and BMI, paragraph 1 |
| Participants | 6 | (*a*) Give the eligibility criteria, and the sources and methods of selection of participants. Describe methods of follow-up | Methods, study cohorts and statistical analyses, paragraph 1 |
| | | (*b*) For matched studies, give matching criteria and number of exposed and unexposed | |
| Variables | 7 | Clearly define all outcomes, exposures, predictors, potential confounders, and effect modifiers. Give diagnostic criteria, if applicable | Methods, "Assisted reproductive technologies and comparison cohorts", "Overweight, obesity and Body Mass Index", "Covariates" |
| Data sources/ measurement | 8* | For each variable of interest, give sources of data and details of methods of assessment (measurement). Describe comparability of assessment methods if there is more than one group | Methods, setting and data sources |
| Bias | 9 | Describe any efforts to address potential sources of bias | Methods, sensitivity analyses |
| Study size | 10 | Explain how the study size was arrived at | Methods, study |

| | | | |
|---|---|---|---|
| | | | cohorts, paragraph 1 |
| Quantitative variables | 11 | Explain how quantitative variables were handled in the analyses. If applicable, describe which groupings were chosen and why | Methods, statistical analyses, paragraph 3 |
| Statistical methods | 12 | (*a*) Describe all statistical methods, including those used to control for confounding | Methods, statistical analyses |
| | | (*b*) Describe any methods used to examine subgroups and interactions | Methods, statistical analyses |
| | | (*c*) Explain how missing data were addressed | Methods, table 2 |
| | | (*d*) If applicable, explain how loss to follow-up was addressed | n/a |
| | | (*e*) Describe any sensitivity analyses | Methods, sensitivity analyses |
| **Results** | | | |
| Participants | 13* | (a) Report numbers of individuals at each stage of study—eg numbers potentially eligible, examined for eligibility, confirmed eligible, included in the study, completing follow-up, and analysed | Methods, study cohort Results, paragraph 1 and 2 |
| | | (b) Give reasons for non-participation at each stage | n/a since study cohorts are not invited |
| | | (c) Consider use of a flow diagram | Figure 1 |
| Descriptive data | 14* | (a) Give characteristics of study participants (eg demographic, clinical, social) and information on exposures and potential confounders | Results, paragraph 1 and 2 and Table 1 and 2 |
| | | (b) Indicate number of participants with missing data for each variable of interest | Table 1 |
| | | (c) Summarise follow-up time (eg, average and total amount) | n/a. Fixed to an age span repeated throughout the manuscript |
| Outcome data | 15* | Report numbers of outcome events or summary measures over time | Figures 2-4 |

| | | | |
|---|---|---|---|
| Main results | 16 | (*a*) Give unadjusted estimates and, if applicable, confounder-adjusted estimates and their precision (eg, 95% confidence interval). Make clear which confounders were adjusted for and why they were included | Result sections: "Crude prevalences of overweight and obesity among children age 5 – 8 years", "The associations between fertility treatments and overweight, obesity, and BMI z-scores in children at age 5-8 years", and Figures 2-4 |
| | | (*b*) Report category boundaries when continuous variables were categorized | Supplementary Figures 2-4 |
| | | (*c*) If relevant, consider translating estimates of relative risk into absolute risk for a meaningful time period | |
| Other analyses | 17 | Report other analyses done—eg analyses of subgroups and interactions, and sensitivity analyses | Results section; "The associations between fertility treatments and overweight, obesity, and BMI z-scores in children at age 5-8 years", and Figures 2-4 |
| **Discussion** | | | |
| Key results | 18 | Summarise key results with reference to study objectives | Discussion, paragraph 1 |
| Limitations | 19 | Discuss limitations of the study, taking into account sources of potential bias or imprecision. Discuss both direction and magnitude of any potential bias | Discussion, paragraph 5 |
| Interpretation | 20 | Give a cautious overall interpretation of results considering objectives, limitations, multiplicity of analyses, results from similar studies, and other relevant evidence | Discussion, paragraph 3, 4, 6 and 7 |
| Generalisability | 21 | Discuss the generalisability (external validity) of the study results | Discussion, paragraph 5 and 7 |
| **Other information** | | | |
| Funding | 22 | Give the source of funding and the role of the funders for the present study and, if applicable, for the original study on which the present article is based | According to guidance by Plos Medicine |

*Give information separately for exposed and unexposed groups.

**Note:** An Explanation and Elaboration article discusses each checklist item and gives methodological background and published examples of transparent reporting. The STROBE checklist is best used in conjunction with this article (freely available on the Web sites of PLoS Medicine at http://www.plosmedicine.org/, Annals of Internal Medicine at

http://www.annals.org/, and Epidemiology at http://www.epidem.com/). Information on the STROBE Initiative is available at http://www.strobe-statement.org.
