# Supplementary material for: Overweight or obesity in children born after assisted reproductive technologies in Denmark: A population-based cohort study
Source: PLoS Med. 2023 Dec 19;20(12):e1004324. doi: 10.1371/journal.pmed.1004324 (PMC10729995; doi:10.1371/journal.pmed.1004324)
Supplement: S1 Text — (PDF) [file pmed.1004324.s002.pdf]

Fertility treatments and risk of childhood overweight and obesity in offspring

Date of last draft: 18-12-2020

Project group:

Kristina Laugesen, Department of Clinical Epidemiology, Aarhus University Hospital, Aarhus, Denmark

Katalin Veres, Department of Clinical Epidemiology, Aarhus University Hospital, Aarhus, Denmark

Henrik Toft Sørensen, Department of Clinical Epidemiology, Aarhus University Hospital, Aarhus, Denmark

Yu-Han Chiu, Harvard TH Chan School of Public Health, Boston, US

John Hsu, Deaprtment of Health Care Policy, Harvard Medical School, Boston, US

Sonia Hernandez-Diaz, Harvard TH Chan School of Public Health, Boston, US

Anne Sara Oberg, Harvard TH Chan School of Public Health, Boston, US

Paolo Rinaudo, School of Medicine, University of California, San Fransisco, US

Mandy Spaan, Netherlands Cancer Institute, Amsterdam, The Netherlands

Flora Van Leeuwen, Netherlands Cancer Institute, Amsterdam, The Netherlands

Amendments were performed post hoc, including use of inverse probability of treatment weights for control of confounding, inclusion of additional potential confounders, and some extra sensitivity analyses.

BACKGROUND

Infertility is a growing public health concern and around 10% of all births occur after use of assisted reproductive technologies (ART) or non-in vitro fertility treatments (NIFT) in Denmark.[1] Short- and long-term outcomes in these children are therefore crucial to investigate, including the impact on metabolic health.

AIM

1) To quantify the effect of ART compared to NIFT on overweight and obesity in offspring at age 5-8 years of age.

2) To investigate if a potential effect differs according to treatment procedure and underlying cause of infertility.

METHODS

Setting: Denmark.

Design: Cohort study.

Data sources:

- *The Danish Medical Birth Register*[2]
- *The Danish IVF Registry*[3]
- *The Danish National Children´s database*
- *The Danish National Patient Registry*[4]
- *The Danish National Prescription Registry*[5]

- *The social and demographic registers in Statistics Denmark*
- *The Civil Registration System*[6]

Study population:

The study population will consist of all children born alive in Denmark between 1 January 2007 and 31 December 2012 identified in the Danish Medical Birth Register[2] (≈ 500,000 children).

Exposure and comparison cohorts

Exposure and comparison cohorts will be established through linkage between the Danish Medical Birth Registry and the Danish IVF Registry and will be defined as below.

- *Children born after ART treatment:* Children born after ART treatment will be identified through a maternal cpr number in the IVF registry up to 12 months before date of birth of the child in combination with the relevant treatment code (Appendix 1).
- *Children born after NIFT treatment:* Children born after NIFT treatment will be identified through a maternal cpr number in the IVF registry up to 12 months before date of birth of the child in combination with the relevant treatment code (Appendix 1).
- *Naturally conceived children:* Naturally conceived children will be identified in the Danish Medical Birth Registry and defined as absence of a maternal cpr number in the IVF registry up to 12 months before date of birth (i.e not born after NIFT or ART treatment).

Outcome

We will use the Danish National Children´s Database to obtain information on height, weight and body mass index (BMI) of children in our study population at time of enrolment in primary school (defined as children aged ≤5 and <8 years at BMI measurement). BMI will be calculated as weight in kilograms divided by the height in metres squared. We will assess BMI as a categorical and continuous outcome, respectively.

- As regards BMI as a categorical outcome we use the Extended International (IOTF) Body Mass Index Cut-Offs to define overweight and obesity (Appendix 1).[7]
- As regards BMI as a continuous outcome we will use the World Health Organization (WHO) 2007 reference data to obtain z-scores (Appendix 1).[8]

Statistical analysis

1) We will describe baseline characteristics in naturally, ART and NIFT concieved children (Table 1). For definitions, please see Appendix 1. Characteristics will include birth year (2007-2009, 2010-2021), cause of infertility/subfertility (female factor any, anovulation/PCOS, tubal factor, endometriosis, umspecified, male factor, idiopathic), index treatment (intra cytoplasmatic sperm injection (ICSI), fresh embryo transfer, frozen embryo transfer, use of partner sperm, use of donor sperm), previous maternal number of procedures involving NIFT (0, 1, 2, ≥3), previous maternal number of procedures involving ART (0, 1, 2, ≥3), maternal age at conception (median and IQR), maternal highest educational level at conception [low (primary and lower secondary education), medium (upper secondary education or academy profession degree), high (university education at bachelor's degree level or higher), missing], maternal pre-pregnancy BMI

(continous and categorical: <18.5, 18.5-24, 25-29, ≥30, missing), maternal smoking during pregnancy (yes, no, missing), maternal diabetes (type I, II) any time before, paternal age at conception, paternal highest educational level at conception, and paternal BMI (only available from 2010 and onwards).

2) We will further describe the study population according to birth and infant characteristics (Table 2). Characteristics include birth order (1, ≥2), gestational age at birth (< 28, 28-31, 32-36, ≥ 37 weeks), birth weight (continous, missing), small for gestational age (yes/no), large for gestational age (yes/no), Apgar score after 5 minutes (<7, 7-10, missing), cesarean section (yes/no).
3) We will examine the association between ARTand BMI compared to NIFT. For the categorical outcomes we will use generalized estimating equations to estimate crude and adjusted relative risks (RR) and risk differences (RD) for overweight and obesity comparing ART concieved children with NIFT (Table 3). For the continous outcomes (BMI Z-scores) we will display the BMI z-scores distributions graphically and further use mariginal generalized estimating equations to estimate crude and adjusted mean differences in BMI z-scores (Table 4). The adjusted models will include: birth year (2007-2009, 2010-2012), birth order (1, ≥2), maternal age at conception (cubic spline with 3 knots), highest maternal educational level at conception (low, medium, high), maternal pre-pregnancy BMI (cubic spline with 3 knots) , maternal smoking during pregnancy (yes, no), maternal diabetes any time before preganancy (type I, II), paternal age at conception (cubic spline with 3 knots), highest paternal educational level at conception (low, medium, high).

Sub analyses

4) We will stratify by sex of the child.

5) We will examine different ART technologies separately, e.g. ICSI vs. ordinary IVF frozen vs. fresh embryo transfer in a study population restricted to children concieved by ART overall and within treatment indication (Table 5 and 6).
6) Depending on our findings in the main analysis, we will explore feasibility of a sibling design.

Sensitivity analyses

7) As we will be unable to adjust for paternal BMI (a potential confounder), we will perform a restricted analysis. The study population will be restricted to children concieved from 1 January 2010 to 31 December 2012 and couples will be excluded if either the woman or the partner have BMI ≥25 kg/m^2.

TABLES

Table 1. Table 1. Baseline characteristics among children born in Denmark from 2007-2012 and who underwent routine anthropometric evaluation at 5-8 years of age.

	Naturally conceived children	Children born after fertility treatment					
		ART					NIFT
		All ART	Ordinary IVF	ICSI	Fresh embryo	Frozen embryo	
Total							
Birth year							
2007-2009							
2010-2012							
Cause of infertility/subfertility							
Female factor (any)							
Anovulation/PCOS							
Tubal factor							
Endometriosis							
Unspecified							
Male factor (any)							
Idiopathic							
Use of partner sperm	NA						
Use of donor sperm	NA						
Previous maternal number of procedures involving NIFT							
0							
1							
2							
≥3							
Previous maternal number of procedures involving ART							
0							
1							
2							
≥3							
Maternal characteristics							

Age at conception (years), median and IQR							
Highest educational level							
Low							
Medium							
High							
Missing							
Pre-pregnancy body mass index (kg/m^2), median IQR							
<18.5							
18.5-24							
25-29							
≥ 30							
Missing							
Smoking during pregnancy							
Yes							
Missing							
Diabetes before pregnancy (type I, II)							
Paternal characteristics							
Age at conception, median and IQR							
Highest educational level at conception							
Low							
Medium							
High							
Missing							
Paternal BMI (kg/m^2)							
<18.5	NA						
18.5-24	NA						
25-29	NA						
≥30	NA						
Missing in the calendar years ≥ 2010	NA						
Missing before 2010 (not recorded)	NA						

Table 2. Descriptive birth characteristics of liveborn children in Denmark, 2007-2012.

	Naturally conceived children	Children born after fertility treatment	
		ART	NIFT
All births			
Single birth			
Twin birth			
Triple birth			
Sex			
Boy			
Girl			
Birth order			
1			
≥2			
Gestational age (weeks)			
< 28			
28 to 31			
32 to 36			
≥ 37			
Birth weight, median (IQR)			
Missing			
Small for gestational age (SGA)			
Large for gestational age (LGA)			
Apgar score after 5 minutes			
<7			
7-10			
Missing			
Caesarean section			

Table 3. The association between fertility treatment and overweigth and obesity at enrollment in primary school overall and within different treatment indications.

	N at risk	N (overweight/ obese)	Relative risk and 95% CI		Risk difference and 95% CI	
			Crude	Adjusted*	Crude	Adjusted*
**All treatment indications**						
**Overweight/obese**						
NIFT			Ref	Ref	Ref	Ref
ART						
**Overweight**						
NIFT			Ref	Ref	Ref	Ref
ART						
**Obese**						
NIFT			Ref	Ref	Ref	Ref
ART						
**Female factor (all)**						
**Overweight/obese**						
NIFT			Ref	Ref	Ref	Ref
ART						
**Overweight**						
NIFT			Ref	Ref	Ref	Ref
ART						
**Obese**						
NIFT			Ref	Ref	Ref	Ref
ART						
**Anovulation/PCOS**						
**Overweight/obese**						
NIFT			Ref	Ref	Ref	Ref
ART						
**Overweight**						

NIFT			Ref	Ref	Ref	Ref
ART						
**Obese**						
NIFT			Ref	Ref	Ref	Ref
ART						
**Tubal factor**						
**Overweight/obese**						
NIFT			Ref	Ref	Ref	Ref
ART						
**Overweight**						
NIFT			Ref	Ref	Ref	Ref
ART						
**Obese**						
NIFT			Ref	Ref	Ref	Ref
ART						
**Endometriosis**						
**Overweight/obese**						
NIFT			Ref	Ref	Ref	Ref
ART						
**Overweight**						
NIFT			Ref	Ref	Ref	Ref
ART						
**Obese**						
NIFT			Ref	Ref	Ref	Ref
ART						
**Female factor, unspecified**						
**Overweight/obese**						
NIFT			Ref	Ref	Ref	Ref
ART						
**Overweight**						
NIFT			Ref	Ref	Ref	Ref
ART						

**Obese**						
NIFT			Ref	Ref	Ref	Ref
ART						
**Male factor (all)**						
**Overweight/obese**						
NIFT			Ref	Ref	Ref	Ref
ART						
**Overweight**						
NIFT			Ref	Ref	Ref	Ref
ART						
**Obese**						
NIFT			Ref	Ref	Ref	Ref
ART						
**Idiopathic**						
**Overweight/obese**						
NIFT			Ref	Ref	Ref	Ref
ART						
**Overweight**						
NIFT			Ref	Ref	Ref	Ref
ART						
**Obese**						
NIFT			Ref	Ref	Ref	Ref
ART						

*Adjusted for: Birth year (2007-2009, 2010-2012), birth order (1, ≥2), maternal age at comception (cubic spline with 3 knots), highest maternal educational level at conception (low, medium, high), maternal pre-pregnancy BMI (cubic spline with 3 knots), maternal smoking during pregnancy (yes, no), maternal diabetes (type I, II), paternal age at conception (cubic spline with 3 knots), highest paternal educational level at conception (low, medium, high)

Table 4. The association between fertility treatment and mean BMI z-scores at enrollment in primary school overall and within different treatment indications.

	**Mean BMI z score differences and 95% CI**	
	**Crude**	**Adjusted***
**All treatment indications**		
NIFT	Ref	Ref
ART		
**Female factor (all)**		
NIFT	Ref	Ref
ART		
**Anovulation/PCOS**		
NIFT	Ref	Ref
ART		
**Tubal factor**		
NIFT	Ref	Ref
ART		
**Endometriosis**		
NIFT	Ref	Ref
ART		
**Female factor, unspecified**		
NIFT	Ref	Ref
ART		
**Male factor (all)**		
NIFT	Ref	Ref
ART		
**Idiopathic**		
NIFT	Ref	Ref
ART		

*Adjusted for: Birth year (2007-2009, 2010-2012), birth order (1, ≥2), maternal age at conception (cubic spline with 3 knots), highest maternal educational level at conception (low, medium, high), maternal pre-pregnancy BMI (cubic spline with 3 knots), maternal smoking during pregnancy (yes, no), maternal diabetes (type I, II), paternal age at conception (cubic spline with 3 knots), highest paternal educational level at birth (low, medium, high)

Table 5. The association between fertility treatment and overweigth and obesity at enrollment in primary school (study population restricted to children concieved by ART) overall and within different treatment indications.

	N at risk	N (overweight/ obese)	Relative risk and 95% CI		Risk difference and 95% CI	
			Crude	Adjusted*	Crude	Adjusted*
**All treatment indications**						
**Overweight/obese**						
Fresh embryo transfer			Ref	Ref	Ref	Ref
Frozen embryo transfer						
**Overweight**						
Fresh embryo transfer			Ref	Ref	Ref	Ref
Frozen embryo transfer						
**Obese**						
Fresh embryo transfer			Ref	Ref	Ref	Ref
Frozen embryo transfer						
**Overweight/obese**						
Ordinary IVF			Ref	Ref	Ref	Ref
ICSI						
**Overweight**						
Ordinary IVF			Ref	Ref	Ref	Ref
ICSI						
**Obese**						
Ordinary IVF			Ref	Ref	Ref	Ref
ICSI						
**Female factor (any)**						
**Overweight/obese**						
Fresh embryo transfer			Ref	Ref	Ref	Ref
Frozen embryo transfer						
**Overweight**						
Fresh embryo transfer			Ref	Ref	Ref	Ref

Frozen embryo transfer						
**Obese**						
Fresh embryo transfer			Ref	Ref	Ref	Ref
Frozen embryo transfer						
**Overweight/obese**						
Ordinary IVF			Ref	Ref	Ref	Ref
ICSI						
**Overweight**						
Ordinary IVF			Ref	Ref	Ref	Ref
ICSI						
**Obese**						
Ordinary IVF			Ref	Ref	Ref	Ref
ICSI						
**Anovulation/PCOS**						
**Overweight/obese**						
Fresh embryo transfer			Ref	Ref	Ref	Ref
Frozen embryo transfer						
**Overweight**						
Fresh embryo transfer			Ref	Ref	Ref	Ref
Frozen embryo transfer						
**Obese**						
Fresh embryo transfer			Ref	Ref	Ref	Ref
Frozen embryo transfer						
**Overweight/obese**						
Ordinary IVF			Ref	Ref	Ref	Ref
ICSI						
**Overweight**						
Ordinary IVF			Ref	Ref	Ref	Ref
ICSI						
**Obese**						
Ordinary IVF			Ref	Ref	Ref	Ref
ICSI						

**Tubal factor**						
**Overweight/obese**						
Fresh embryo transfer			Ref	Ref	Ref	Ref
Frozen embryo transfer						
**Overweight**						
Fresh embryo transfer			Ref	Ref	Ref	Ref
Frozen embryo transfer						
**Obese**						
Fresh embryo transfer			Ref	Ref	Ref	Ref
Frozen embryo transfer						
**Overweight/obese**						
Ordinary IVF			Ref	Ref	Ref	Ref
ICSI						
**Overweight**						
Ordinary IVF			Ref	Ref	Ref	Ref
ICSI						
**Obese**						
Ordinary IVF			Ref	Ref	Ref	Ref
ICSI						
**Endometriosis**						
**Overweight/obese**						
Fresh embryo transfer			Ref	Ref	Ref	Ref
Frozen embryo transfer						
**Overweight**						
Fresh embryo transfer			Ref	Ref	Ref	Ref
Frozen embryo transfer						
**Obese**						
Fresh embryo transfer			Ref	Ref	Ref	Ref
Frozen embryo transfer						
**Overweight/obese**						
Ordinary IVF			Ref	Ref	Ref	Ref
ICSI						

**Overweight**						
Ordinary IVF			Ref	Ref	Ref	Ref
ICSI						
**Obese**						
Ordinary IVF			Ref	Ref	Ref	Ref
ICSI						
**Female factor unspecified**						
**Overweight/obese**						
Fresh embryo transfer			Ref	Ref	Ref	Ref
Frozen embryo transfer						
**Overweight**						
Fresh embryo transfer			Ref	Ref	Ref	Ref
Frozen embryo transfer						
**Obese**						
Fresh embryo transfer			Ref	Ref	Ref	Ref
Frozen embryo transfer						
**Overweight/obese**						
Ordinary IVF			Ref	Ref	Ref	Ref
ICSI						
**Overweight**						
Ordinary IVF			Ref	Ref	Ref	Ref
ICSI						
**Obese**						
Ordinary IVF			Ref	Ref	Ref	Ref
ICSI						
**Male factor (any)**						
**Overweight/obese**						
Fresh embryo transfer			Ref	Ref	Ref	Ref
Frozen embryo transfer						
**Overweight**						
Fresh embryo transfer			Ref	Ref	Ref	Ref
Frozen embryo transfer						

**Obese**						
Fresh embryo transfer			Ref	Ref	Ref	Ref
Frozen embryo transfer						
**Overweight/obese**						
Ordinary IVF			Ref	Ref	Ref	Ref
ICSI						
**Overweight**						
Ordinary IVF			Ref	Ref	Ref	Ref
ICSI						
**Obese**						
Ordinary IVF			Ref	Ref	Ref	Ref
ICSI						
**Idiopathic**						
**Overweight/obese**						
Fresh embryo transfer			Ref	Ref	Ref	Ref
Frozen embryo transfer						
**Overweight**						
Fresh embryo transfer			Ref	Ref	Ref	Ref
Frozen embryo transfer						
**Obese**						
Fresh embryo transfer			Ref	Ref	Ref	Ref
Frozen embryo transfer						
**Overweight/obese**						
Ordinary IVF			Ref	Ref	Ref	Ref
ICSI						
**Overweight**						
Ordinary IVF			Ref	Ref	Ref	Ref
ICSI						
**Obese**						
Ordinary IVF			Ref	Ref	Ref	Ref
ICSI						

*Adjusted for: Birth year (2007-2009, 2010-2013), birth order (1, ≥2), maternal age at conception (cubic spline with 3 knots), highest maternal educational level at conception (low, medium, high), maternal pre-pregnancy BMI (cubic spline with 3 knots), maternal smoking during pregnancy (yes, no), maternal diabetes (type I, II), paternal age at conception (cubic spline with 3 knots), highest paternal educational level at conception (low, medium, high)

Table 6. The association between fertility treatment and mean BMI z-scores at enrollment in primary school (study population restricted to children concieved by ART) overall and within different treatment indications.

	Mean BMI z score differences and 95% CI	
	**Crude**	**Adjusted***
**All treatment indications**		
Fresh embryo tranfer	Ref	Ref
Frozen embryo transfer		
Ordinary IVF	Ref	Ref
ICSI		
**Female factor (any)**		
Fresh embryo tranfer	Ref	Ref
Frozen embryo transfer		
Ordinary IVF	Ref	Ref
ICSI		
**Anovulation/PCOS**		
Fresh embryo tranfer	Ref	Ref
Frozen embryo transfer		
Ordinary IVF	Ref	Ref
ICSI		
**Tubal factor**		
Fresh embryo tranfer	Ref	Ref
Frozen embryo transfer		
Ordinary IVF	Ref	Ref
ICSI		
**Endometriosis**		
Fresh embryo tranfer	Ref	Ref
Frozen embryo transfer		
Ordinary IVF	Ref	Ref
ICSI		
**Female factor, unspecified**		
Fresh embryo tranfer	Ref	Ref
Frozen embryo transfer		
Ordinary IVF	Ref	Ref
ICSI		
**Male factor**		
Fresh embryo tranfer	Ref	Ref
Frozen embryo transfer		
Ordinary IVF	Ref	Ref
ICSI		
**Idiopathic**		
Fresh embryo tranfer	Ref	Ref
Frozen embryo transfer		
Ordinary IVF	Ref	Ref
ICSI		

*Adjusted for: Birth year (2007-2009, 2010-2012), birth order (1, ≥2), maternal age at conception (cubic spline with 3 knots), highest maternal educational level at conception (low, medium, high), maternal pre-pregnancy BMI (cubic spline with 3 knots), maternal smoking during pregnancy (yes, no), maternal diabetes (type I, II), paternal age at conception (cubic spline with 3 knots), highest paternal educational level at birth (low, medium, high)

REFERENCES

1. Cui L, Zhou W, Xi B, et al. Increased risk of metabolic dysfunction in children conceived by assisted reproductive technology. *Diabetologia* 2020.
2. Bliddal M, Broe A, Pottegard A, Olsen J, Langhoff-Roos J. The Danish Medical Birth Register. *Eur J Epidemiol* 2018; **33**(1): 27-36.
3. Blenstrup LT, Knudsen LB. Danish registers on aspects of reproduction. *Scand J Public Health* 2011; **39**(7 Suppl): 79-82.
4. Schmidt M, Schmidt SA, Sandegaard JL, Ehrenstein V, Pedersen L, Sorensen HT. The Danish National Patient Registry: a review of content, data quality, and research potential. *Clinical epidemiology* 2015; **7**: 449-90.
5. Pottegard A, Schmidt SA, Wallach-Kildemoes H, Sorensen HT, Hallas J, Schmidt M. Data Resource Profile: The Danish National Prescription Registry. *International journal of epidemiology* 2016.
6. Schmidt M, Pedersen L, Sorensen HT. The Danish Civil Registration System as a tool in epidemiology. *European journal of epidemiology* 2014; **29**(8): 541-9.
7. WorldObesity. Obesity Classification. https://www.worldobesity.org/about/about-obesity/obesity-classification (accessed 06/12 2020).
8. WHO. Growth reference data for 5-19 years. https://www.who.int/toolkits/growth-reference-data-for-5to19-years/application-tools (accessed 06/12 2020).
