# Supplementary material for: Overweight or obesity in children born after assisted reproductive technologies in Denmark: A population-based cohort study
Source: PLoS Med. 2023 Dec 19;20(12):e1004324. doi: 10.1371/journal.pmed.1004324 (PMC10729995; doi:10.1371/journal.pmed.1004324)
Supplement: S2 Text — (PDF) [file pmed.1004324.s003.pdf]

	N (%)	
	**With anthropometric evaluation**	**Without anthropometric evaluation**
**Total**	327,301 (100)	49,465 (100)
Born after ART	13,675 (4.2)	2,131 (4.3)
Born after OI/IUI	7,728 (2.4)	1,117 (2.3)
Year of conception		
2006-2009	222,903 (68.1)	20,701 (41.8)
2010-2012	104,398 (31.9)	28,764 (58.2)
Maternal parity		
0	143,361 (43.8)	22,294 (45.1)
≥1	171,808 (52.5)	26,734 (54.0)
Missing	12,132 (3.7)	437 (0.9)
Parental infertility		
Female factor (any)	46,584 (14.2)	6,736 (13.6)
Ovulation disorders	9,128 (2.8)	1,321 (2.7)
Tubal factor	11,280 (3.4)	1,612 (3.3)
Cervical or uterine factors	11,005 (3.4)	1,671 (3.4)
Nonspecific female factor	12,969 (4.0)	1,796 (3.6)
Male factor (any)	21,049 (6.4)	2,998 (6.1)
Idiopathic	789 (0.2)	161 (0.3)
Previous cycles of OI/IUI		
0	311,094 (95.0)	46,901 (94.8)
1	4,851 (1.5)	767 (1.6)
2	3,639 (1.1)	543 (1.1)
>=3	7,717 (2.4)	1,254 (2.5)
Previous cycles of ART		
0	312,948 (95.6)	47,120 (95.3)
1	5,310 (1.6)	782 (1.6)
2	3,584 (1.1)	536 (1.1)
>=3	5,459 (1.7)	1,027 (2.1)
Maternal characteristics		
Age at conception (years), median and IQR	30 (27-34)	30 (27-34)
Highest educational level		
Low	53,290 (16.3)	9,289 (18.8)
Medium	132,969 (40.6)	18,860 (38.1)
High	129,684 (39.6)	16,930 (34.2)
Missing	11,358 (3.5)	4,386 (8.9)
Country of origin		
Nordic	278,916 (85.2)	35,270 (71.3)
European (except the Nordic countries)	15,967 (4.9)	2,470 (5.0)
Non-European	27,707 (8.5)	3,939 (8.0)
Missing/unknown	4,711 (1.4)	7,786 (15.7)
BMI at start pregnancy (kg/m^2), median, IQR	23 (21-26)	23 (21-27)
Smoking during pregnancy		
Yes	40,434 (12.4)	6,837 (13.8)
Diabetes (Type I, II)	7,079 (2.2)	1,163 (2.4)
Hyperlipidaemia/lipid-modifying drugs	1,708 (0.5)	273 (0.6)
Hypertension/antihypertensive drugs	35,241 (10.8)	5,047 (10.2)
Paternal characteristics		
Age at conception, median and IQR	33 (29-37)	33 (29-37)
Highest educational level		
Low	56,915 (17.4)	9,004 (18.2)
Medium	166,113 (50.8)	23,318 (47.1)
High	86,920 (26.6)	11,376 (23.0)
Missing	17,353 (5.3)	5,767 (11.7)
Hyperlipidaemia/lipid-modifying drugs	4,062 (1.2)	696 (1.4)

Hypertension/antihypertensive drugs	15,609 (4.8)	2,433 (4.9)
Birth outcomes		
Child born after singleton pregnancy	313,715 (95.8)	47,015 (95.0)
Gestational age (weeks)		
<28	713 (0.2)	720 (1.5)
28-31	2,387 (0.7)	599 (1.2)
32-36	10,828 (3.3)	1,827 (3.7)
37+	305,927 (93.5)	46,265 (93.5)
Missing	7,446 (2.3)	54 (0.1)
SGA	30,981 (9.5)	5,361 (10.8)
LGA	32,790 (10.0)	4,487 (9.1)
Apgar score at 5 minutes		
7-10	316,486 (96.7)	48,042 (97.1)
<7	2,184 (0.7)	978 (2.0)
Missing	8,631 (2.6)	445 (0.9)
Caesarean section	72,066 (22.0)	11,461 (23.2)

Abbreviations: ART, assisted reproductive technologies; BMI, body mass index; IQR, interquartile range; IUI, intrauterine insemination LGA, large for gestational age; OI, ovulation induction; SGA, small for gestational age.
