# Supplementary material for: Overweight or obesity in children born after assisted reproductive technologies in Denmark: A population-based cohort study
Source: PLoS Med. 2023 Dec 19;20(12):e1004324. doi: 10.1371/journal.pmed.1004324 (PMC10729995; doi:10.1371/journal.pmed.1004324)
Supplement: S3 Text — (PDF) [file pmed.1004324.s004.pdf]

Covariate	Data source	Codes
Female factor (any) before conception	Fertility Treatment Registry or DNPR	ICD-10:<br>N970, N971, N972, N978, N979, E282, N80, N91, E28, N70, N71, N72, N972, N973, N838B, O00, N840, N841, N85, D25, Q51
Ovulation disorders before conception	Fertility Treatment Registry or DNPR	ICD-10:<br>N970, E282, N91, E28, N838B
Tubal factor before conception	Fertility Treatment Registry or DNPR	ICD-10:<br>N971, N70, O00
Cervival or uterine factors before conception	Fertility Treatment Registry or DNPR	ICD-10:<br>N80, N71, N72, N972, N973, N840, N841, N85, D25, Q51
Nonspecific female factor before conception	Fertility Treatment Registry or DNPR	ICD-10:<br>N979 and no prior records of other female factors
Male factor (any) before conception	Fertility Treatment Registry or DNPR	ICD-10:<br>N469, N974, E29
Idiopathic	Fertility Treatment Registry	b_idiopatisk= 1 at index treatment and no prior records of any female factor or male factor
Intracytoplasmic sperm injection (ICSI)	Fertility Treatment Registry	c_in_vitro_metode="ICSI" or<br>c_in_vitro_metode="IVF+ICSI"<br>c_in_vitro_metode="TTP"
Fresh embryo transfer	Fertility Treatment Registry	c_behandling="ALM"
Frozen-thawed embryo transfer	Fertility Treatment Registry	c_behandling="FER"
Diabetes before conception	DNPR and Prescription Registry	ICD-8:<br>249, 250<br>ICD-10:<br>E10-E14, G632, H360, N083<br>If a woman with PCOS fullfilled the criteria only by use of metformin (i.e. had no hospital record of diabetes or use of other antidiabetic treatment), she was recoded as not having diabetes.
Hyperlipidaemia/lipid-modifying drugs before conception	DNPR and Prescription Registry	ICD-8: 27900, 27901<br>ICD-10: E78<br>ATC code: C10
Hypertension/antihypertensive drugs before conception	DNPR and Prescription Registry	ICD-8: 400-404<br>ICD-10: I10-I15<br>ATC code: C02-C04, C07-C09

Abbreviations: ATC, Anatomical Therapeutic Chemical code; DNPR, Danish National Patient Registry; ICD, *International Classification of Diseases*; IVF Registry: In vitro fertilization Registry
