# Supplementary material for: Overweight or obesity in children born after assisted reproductive technologies in Denmark: A population-based cohort study
Source: PLoS Med. 2023 Dec 19;20(12):e1004324. doi: 10.1371/journal.pmed.1004324 (PMC10729995; doi:10.1371/journal.pmed.1004324)
Supplement: S4 Text — (PDF) [file pmed.1004324.s005.pdf]

	Crude POR (95% CI)		Adjusted* POR (95% CI)	
	**Overweight**	**Obesity**	**Overweight**	**Obesity**
Year of conception				
2006-2009	Ref	Ref	Ref	Ref
2010-2012	1.04 (1.02–1.07)	1.10 (1.05–1.15)	1.06 (1.03–1.09)	1.10 (1.05–1.16)
Parental cause of infertility				
Female factor (any)	1.04 (1.01–1.07)	1.08 (1.01–1.14)	1.02 (0.95–1.11)	0.99 (0.83–1.17)
Ovulation disorders	1.23 (1.16–1.30)	1.49 (1.34–1.66)	1.14 (1.05–1.25)	1.32 (1.11–1.58)
Tubal factor	1.10 (1.04–1.16)	1.15 (1.03–1.29)	1.05 (0.96–1.14)	1.06 (0.89–1.27)
Cervical or uterine factors	0.99 (0.94–1.05)	0.96 (0.85–1.09)	1.00 (0.91–1.09)	0.96 (0.80–1.15)
Nonspecific female factor	0.99 (0.93–1.05)	1.03 (0.91–1.16)	1.00 (0.91–1.09)	1.06 (0.89–1.27)
Male factor (any)	0.86 (0.82–0.90)	0.80 (0.72–0.88)	0.96 (0.91–1.02)	0.93 (0.82–1.05)
Idiopathic	0.72 (0.57–0.92)	0.62 (0.36–1.07)	0.83 (0.64–1.07)	0.76 (0.41–1.40)
Previous OI/IUI cycles				
0	Ref	Ref	Ref	Ref
1	0.88 (0.81–0.97)	0.80 (0.66–0.97)	0.99 (0.90–1.10)	0.93 (0.75–1.16)
2	0.88 (0.80–0.98)	0.84 (0.68–1.05)	0.98 (0.87–1.10)	1.01 (0.79–1.29)
>=3	0.79 (0.74–0.85)	0.70 (0.60–0.83)	0.94 (0.86–1.03)	0.96 (0.79–1.17)
Previous ART cycles				
0	Ref	Ref	Ref	Ref
1	0.78 (0.71–0.85)	0.77 (0.64–0.93)	0.88 (0.79–0.98)	0.92 (0.74–1.15)
2	0.75 (0.67–0.83)	0.59 (0.45–0.77)	0.90 (0.79–1.01)	0.80 (0.60–1.07)
>=3	0.79 (0.72–0.86)	0.76 (0.63–0.91)	0.97 (0.87–1.07)	1.07 (0.85–1.33)
Maternal characteristics				
Age at conception (years)				
<25	1.38 (1.34–1.42)	1.49 (1.40–1.59)	1.08 (1.02–1.14)	1.03 (0.92–1.15)
25 - 29	Ref	Ref	Ref	Ref
30 - 34	0.91 (0.89–0.93)	0.88 (0.84–0.93)	0.98 (0.93–1.02)	0.99 (0.90–1.09)
35 - 40	1.02 (0.98–1.05)	1.10 (1.03–1.17)	1.03 (0.95–1.11)	1.12 (0.94–1.32)
>40	1.24 (1.17–1.32)	1.41 (1.25–1.59)	1.11 (0.98–1.27)	1.19 (0.91–1.55)
Parity				
0	Ref	Ref	Ref	Ref
≥1	1.12 (1.09–1.14)	1.26 (1.20–1.31)	1.04 (1.01–1.06)	1.03 (0.98–1.09)
Highest educational level				
Low	2.40 (2.33–2.47)	4.38 (4.12–4.66)	1.33 (1.28–1.38)	1.75 (1.61–1.89)
Medium	1.58 (1.54–1.62)	2.26 (2.13–2.39)	1.15 (1.12–1.18)	1.37 (1.29–1.47)
High	Ref	Ref	Ref	Ref
Country of origin				
Nordic	Ref	Ref	Ref	Ref
European (except Nordic countries)	1.98 (1.90-2.06)	3.08 (2.87-3.29)		2.95 (2.70-3.22)
Non-European	1.65 (1.60-1.71)	2.33 (2.20–2.48)	0.82 (0.77-0.88)	2.22 (2.05-2.40)
BMI at start pregnancy (kg/m^2)				
<18.5	0.48 (0.44–0.52)	0.45 (0.36–0.57)	0.42 (0.38–0.46)	0.40 (0.31–0.51)
18.5-24	Ref	Ref	Ref	Ref
25-29	2.23 (2.18–2.29)	2.88 (2.71–3.05)	2.01 (1.95–2.07)	2.49 (2.33–2.66)
30-34	3.56 (3.45–3.68)	5.69 (5.34–6.07)	2.92 (2.80–3.04)	4.47 (4.13–4.83)
≥ 35	5.58 (5.37–5.81)	11.1 (10.4–11.8)	4.23 (4.01–4.46)	8.14 (7.41–8.94)
Smoking during pregnancy	1.99 (1.94–2.05)	2.74 (2.60–2.87)	1.64 (1.59–1.69)	2.04 (1.93–2.17)
Diabetes (type I, II)	1.89 (1.66–2.14)	2.48 (2.00–3.09)	1.23 (1.05–1.52)	1.43 (1.26–1.63)
Hyperlipidaemia	1.33 (1.05–1.69)	2.25 (1.55–3.27)	1.05 (0.80–1.37)	1.71 (0.90–2.66)
Lipid-modifying drugs	1.74 (1.53–1.97)	2.09 (1.67–2.63)	1.15 (0.99–1.34)	0.95 (0.72–1.25)
Hypertension	1.39 (1.23–1.57)	1.61 (1.28–2.03)	1.11 (0.96–1.27)	1.16 (0.89–1.50)
Antihypertensive drugs	1.15 (1.12–1.19)	1.23 (1.15–1.31)	1.06 (1.02–1.10)	1.05 (0.97–1.13)
Paternal characteristics				
Age at conception (years)				

<25	1.32 (1.27–1.37)	1.46 (1.34–1.58)	1.01 (0.97–1.05)	1.04 (0.93–1.17)
25 – 30	Ref	Ref	Ref	Ref
30 - 35	0.89 (0.87–0.92)	0.89 (0.84–0.95)	1.01 (0.97–1.05)	1.01 (0.92–1.10)
35 – 40	0.96 (0.93–0.99)	1.06 (0.99–1.13)	1.06 (0.99–1.13)	1.06 (0.93–1.22)
>40	1.14 (1.10–1.18)	1.30 (1.21–1.40)	1.10 (0.99–1.22)	0.98 (0.79–1.21)
Highest educational level				
Low	2.65 (2.56–2.73)	5.23 (4.84–5.65)	1.49 (1.43–1.55)	2.08 (1.90–2.28)
Medium	1.74 (1.69–1.79)	2.61 (2.43–2.81)	1.27 (1.23–1.32)	1.57 (1.45–1.70)
High	Ref	Ref	Ref	Ref
Hyperlipidaemia	1.33 (1.05–1.69)	2.25 (1.55–3.27)	0.78 (0.63–0.97)	0.74 (0.50–1.09)
Lipid-modifying drugs	1.55 (1.43–1.69)	2.35 (2.05–2.70)	1.15 (1.34–1.27)	1.47 (1.24–1.75)
Hypertension	1.57 (1.40–1.76)	1.90 (1.54–2.34)	1.17 (1.02–1.34)	1.07 (0.83–1.38)
Antihypertensive drugs	1.35 (1.29–1.41)	1.58 (1.45–1.72)	1.17 (1.11–1.23)	1.21 (1.09–1.34)

*Mutually adjusted for the remaining covariates. Abbreviations: ART, assisted reproductive technologies; BMI, body mass index; CI, confidence interval; IUI, intrauterine insemination; OI, ovulation induction; POR, prevalence odds ratio.
