# Supplementary material for: Overweight or obesity in children born after assisted reproductive technologies in Denmark: A population-based cohort study
Source: PLoS Med. 2023 Dec 19;20(12):e1004324. doi: 10.1371/journal.pmed.1004324 (PMC10729995; doi:10.1371/journal.pmed.1004324)
Supplement: S5 Text — (PDF) [file pmed.1004324.s006.pdf]

	Adjusted POR (95% CI)	
	**Boys**	**Girls**
**ART vs. OI/IUI**		
Overweight	0.97 (0.83–1.13); *p* = 0.72	1.01 (0.89–1.15); *p* = 0.89
Obesity	1.09 (0.75–1.59); *p* = 0.64	0.96 (0.69–1.32); *p* = 0.78
**Frozen-thawed vs. fresh embryo transfer**		
Overweight	1.05 (0.81–1.36); *p* = 0.74	1.06 (0.87–1.29); *p* = 0.55
Obesity	1.88 (1.11–3.20); *p* = 0.02	1.45 (0.91–2.30); *p* =0.11
**ICSI vs. conventional IVF**		
Overweight	1.11 (0.93–1.34); *p* = 0.26	0.87 (0.74–1.02); *p* =0.09
Obesity	1.14 (0.78–1.69); *p* = 0.50	1.00 (0.66–1.51); *p* = 0.28

We adjusted for parental causes of infertility, maternal and paternal age at conception, maternal and paternal highest educational level at conception, maternal country of origin, maternal BMI, maternal smoking status, maternal and paternal hyperlipidemia/use of lipid-modifying drugs, maternal and paternal hypertension/use of antihypertensive drugs, diabetes (type I or II) diagnosed at any time before conception, parity, and year of conception. P-values were calculated by the large-sample Wald (Z) test. Abbreviations: ART, assisted reproductive technologies; CI, confidence interval; ICSI, intracytoplasmic sperm injection; IUI, intrauterine insemination; IVF, in vitro fertilization; OI, ovulation induction; POR, prevalence odds ratio
