# Supplementary material for: Overweight or obesity in children born after assisted reproductive technologies in Denmark: A population-based cohort study
Source: PLoS Med. 2023 Dec 19;20(12):e1004324. doi: 10.1371/journal.pmed.1004324 (PMC10729995; doi:10.1371/journal.pmed.1004324)
Supplement: S6 Text — (PDF) [file pmed.1004324.s007.pdf]

	Adjusted POR (95% CI)							
	**Main analysis**	**Analysis restricted to first ART cycle**	**Previous ART and IUI/OI cycles included in the IPTW**	**Paternal BMI included in the IPTW (sub population[a])**	**Frozen-thawed vs. fresh analysis restricted to conventional IVF**	**ICSI vs. conventional IVF analysis restricted to fresh embryo transfer**	**Frozen-thawed in natural cycle vs. fresh analysis**	**Frozen-thawed in medicated cycle vs. fresh analysis**
**ART vs. NIFT**								
Overweight	1.00 (0.91–1.11); *p* = 0.95	1.01 (0.90–1.15); *p* = 0.82	NA	0.96 (0.72–1.28); *p* = 0.78	NA	NA	NA	NA
Obesity	1.01 (0.79–1.29); *p* = 0.94	1.02 (0.75–1.40); *p* = 0.89	NA	1.01 (0.49–2.07); *p* = 0.48	NA	NA	NA	NA
**Frozen-thawed vs. fresh embryo transfer**								
Overweight	1.08 (0.92–1.26); *p* = 0.36	NA	NA	NA	1.14 (0.86–1.50); *p* = 0.36	NA	0.99 (0.78-1.26); *p* = 0.94	1.05 (0.83-1.32); *p* = 0.69
Obesity	1.54 (1.09–2.17); *p* = 0.01	NA	NA	NA	1.45 (0.82–2.56); *p* = 0.20	NA	1.36 (0.79-2.33); *p* = 0.26	1.46 (0.91-2.35); *p* = 0.12
**ICSI vs. conventional IVF**								
Overweight	0.95 (0.83–1.07); *p* = 0.39	0.97 (0.84–1.10); *p* = 0.61	0.92 (0.81–1.05); *p* = 0.24	NA	NA	0.97 (0.84–1.10); *p* = 0.61	NA	NA
Obesity	1.16 (0.84–1.61); *p* = 0.36	1.29 (0.90–1.84); *p* = 0.16	1.12 (0.81–1.55); *p* = 0.50	NA	NA	1.29 (0.90–1.84); *p* = 0.16	NA	NA

Adjusted for parental causes of infertility, maternal and paternal age at conception, maternal and paternal highest educational level at conception, maternal country of origin, maternal BMI, maternal smoking status, maternal and paternal hyperlipidemia/use of lipid-modifying drugs, maternal and paternal hypertension/use of antihypertensive drugs, diabetes (type I or II) diagnosed at any time before conception, parity, and year of conception.

Abbreviations: ART, assisted reproductive technologies; ICSI, intracytoplasmic sperm injection; IUI, intrauterine insemination; IVF, in vitro fertilization; IPTW, inverse probability of treatment weighting; NA, not assessed; OI, ovulation induction; POR, prevalence odds ratio

[a]Children born during 2011-2012. The percentage of children with missing paternal Body Mass Index was high (60-70%).

P-values were calculated by the large-sample Wald (Z) test.
