# Supplementary material for: Overweight or obesity in children born after assisted reproductive technologies in Denmark: A population-based cohort study
Source: PLoS Med. 2023 Dec 19;20(12):e1004324. doi: 10.1371/journal.pmed.1004324 (PMC10729995; doi:10.1371/journal.pmed.1004324)
Supplement: S7 Text — (PDF) [file pmed.1004324.s008.pdf]

	Crude			Adjusted		
	**N of events/ N at risk**	**Prevalence (%)**	**POR (95% CI)**	**N of events/ N at risk**	**Prevalence (%)**	**POR (95% CI)**
**ART**						
**Overweight**						
General population	33,465/265,101	13	Ref	1355/12,279	11	Ref
ART	1280/12,248	10	0.81 (0.77-0.86)	1280/12,248	10	0.95 (0.90 to 1.01); p = 0.08
**Obesity**						
General population	6803/265,101	3.0	Ref	246/12,279	2.0	Ref
ART	230/12,248	2.0	0.73 (0.64-0.82)	230/12,248	1.9	0.94 (0.82 to 1.07); p = 0.4
**Frozen-thawed embryo transfer**						
**Overweight**						
General population	33,563/265,627	13	Ref	163/1542	11	Ref
Frozen-thawed embryo transfer	169/1553	11	0.84 (0.73-0.97)	7/61	11	1.03 (0.89 to 1.20); p = 0.7
**Obesity**						
General population	6824/265,627	3.0	0.99 (0.74-1.32)	NA	NA	Ref
Frozen-thawed embryo transfer	42/1553	3.0		NA	NA	1.45 (1.07 to 1.96); p= 0.02

Notes: We adjusted for maternal and paternal age at conception, maternal and paternal highest educational level at conception, maternal country of origin, maternal body mass index, maternal smoking status, maternal and paternal hyperlipidemia/use of lipid-modifying drugs, maternal and paternal hypertension/use of antihypertensive drugs, diabetes (type I or II) diagnosed at any time before conception, parity, and year of conception. P-values were calculated by the large-sample Wald (Z) test. Abbreviations: ART, assisted reproductive technologies; CI, confidence interval; NA, not available due to Danish legislation concerning micro data, POR, prevalence odds ratio.
