# Supplementary material for: Overweight or obesity in children born after assisted reproductive technologies in Denmark: A population-based cohort study
Source: PLoS Med. 2023 Dec 19;20(12):e1004324. doi: 10.1371/journal.pmed.1004324 (PMC10729995; doi:10.1371/journal.pmed.1004324)
Supplement: S8 Text — (PDF) [file pmed.1004324.s009.pdf]

	Difference in mean BMI Z-score (95% CI)	
	**Crude**	**Adjusted**
**ART**	-0.14 (-0.16 to 0.12)	-0.08 (-0.10 to -0.06); $p < 0.001$
**Frozen-thawed embryo transfer**	-0.08 (-0.13 to 0.03)	0.00 (-0.06 to 0.05); $p = 0.9$

Notes: We adjusted for maternal and paternal age at conception, maternal and paternal highest educational level at conception, maternal country of origin, maternal BMI, maternal smoking status, maternal and paternal hyperlipidemia/use of lipid-modifying drugs, maternal and paternal hypertension/use of antihypertensive drugs, diabetes (type I or II) diagnosed at any time before conception, parity, and year of conception. P-values were calculated by the large-sample Wald (Z) test. Abbreviations: ART, assisted reproductive technologies; BMI, body mass index; CI, confidence interval.
