# Supplementary material for: Overweight or obesity in children born after assisted reproductive technologies in Denmark: A population-based cohort study
Source: PLoS Med. 2023 Dec 19;20(12):e1004324. doi: 10.1371/journal.pmed.1004324 (PMC10729995; doi:10.1371/journal.pmed.1004324)
Supplement: S1 Fig — Propensity score distributions across exposure and comparison cohorts overall and for subgroups defined by underlying cause of infertility for (A) ARTs vs. OI with or without IUI (OI/IUI), (B) ICSI vs. cIVF, and (C) frozen-thawed vs. fresh embryo transfer. Notes: Abbreviations: ART, assisted reproductive technology; cIVF, conventional in vitro fertilization; ICSI, intracytoplasmic sperm injection; IUI, intrauterine insemination; OI; ovulation induction. (PDF) [file pmed.1004324.s010.pdf]

(A) Assisted reproductive technologies (ART) vs. ovulation induction with or without intrauterine insemination (OI/IUI)

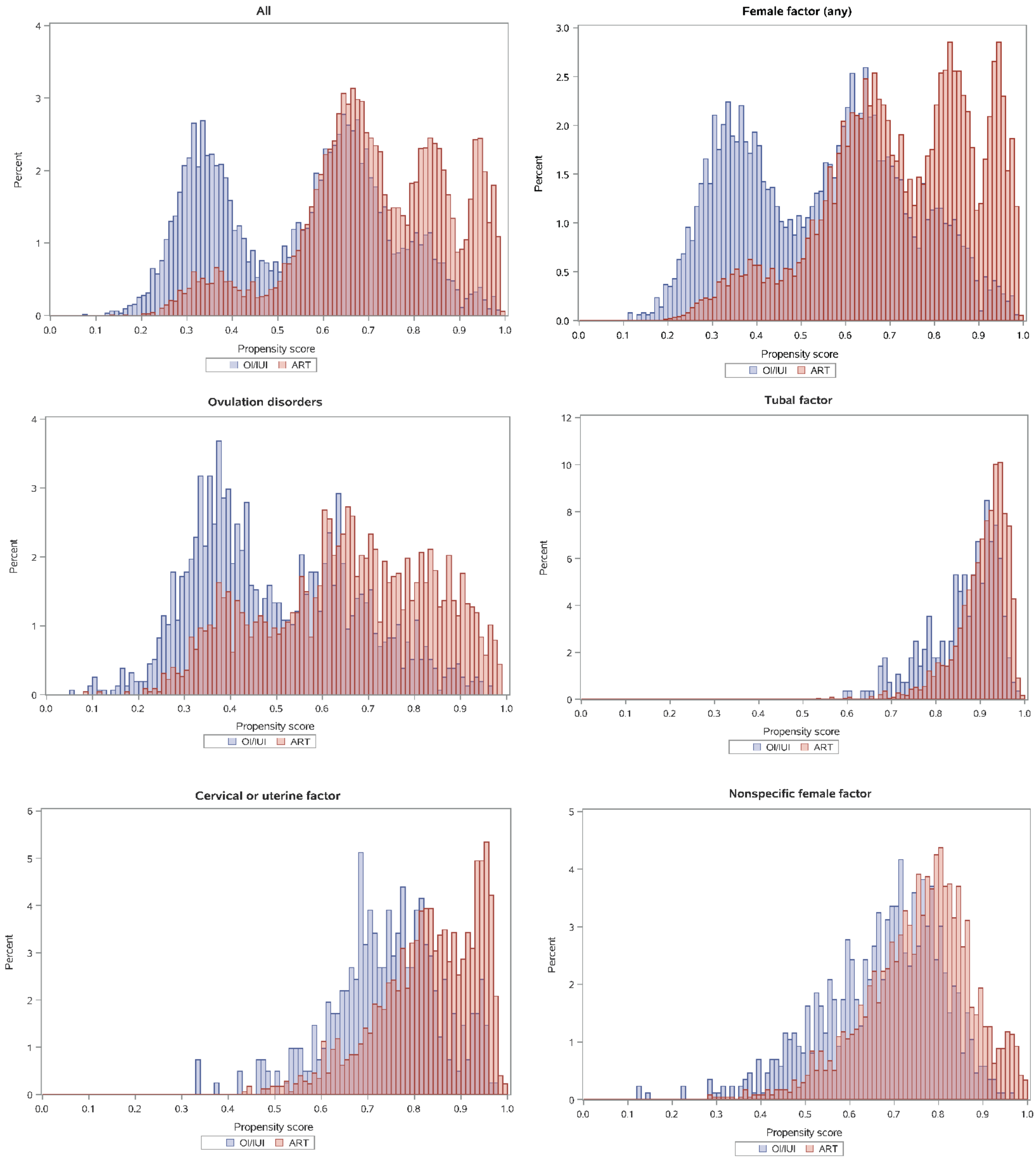

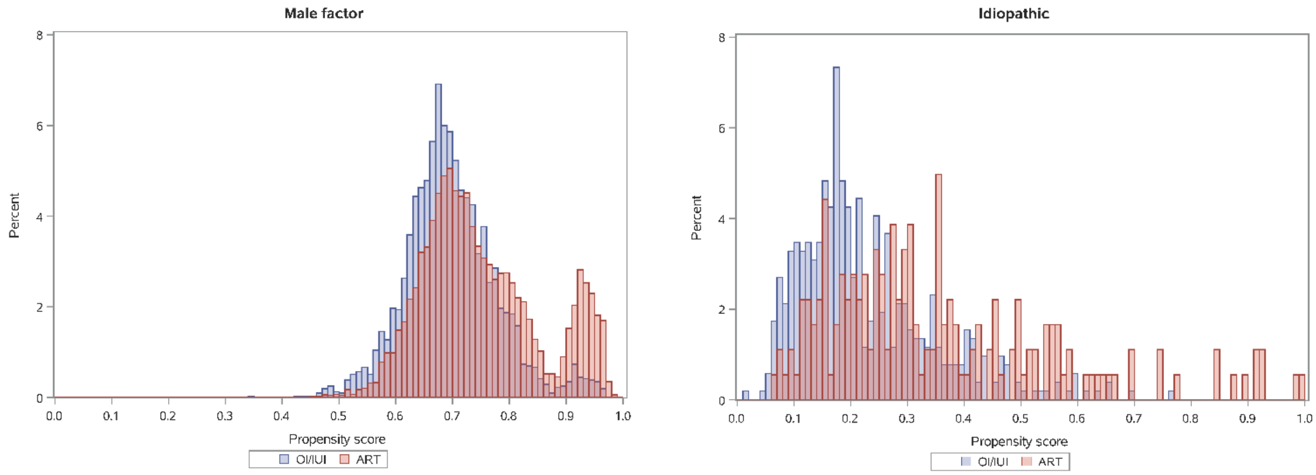

(B) Intracytoplasmic sperm injection (ICSI) vs. conventional in vitro fertilization (cIVF)

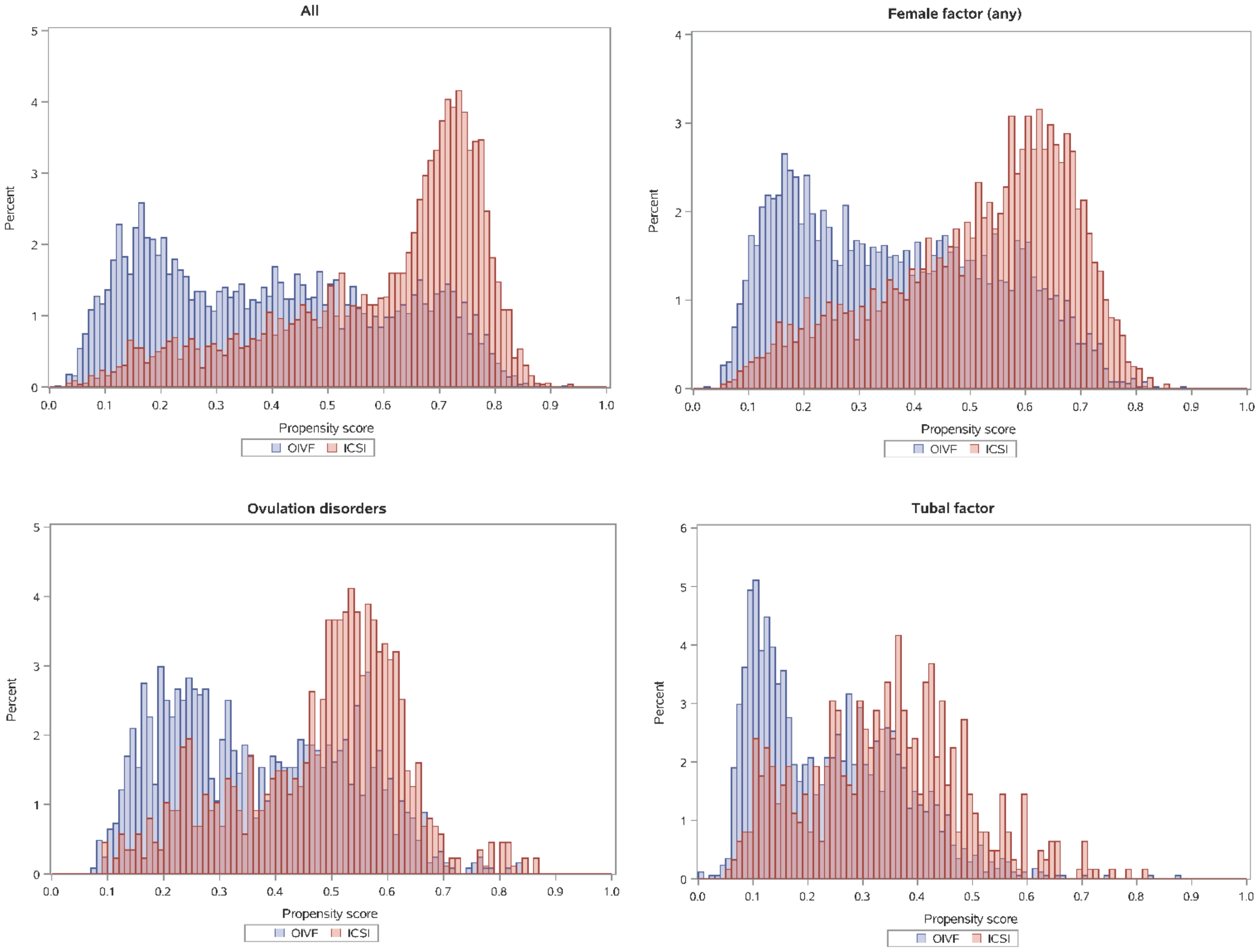

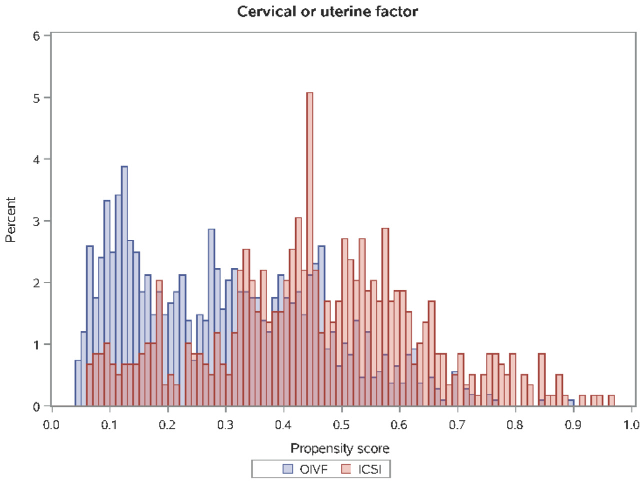

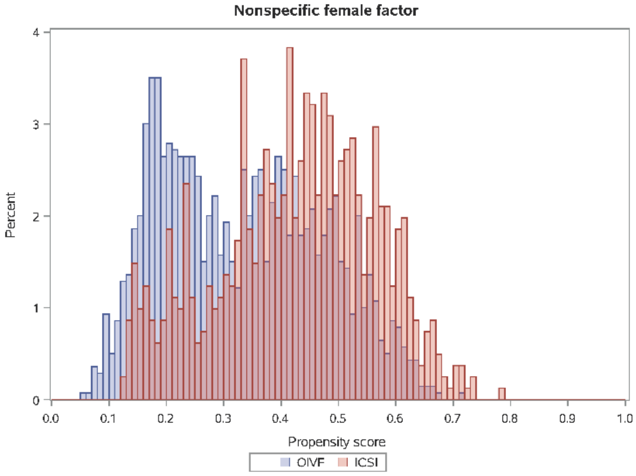

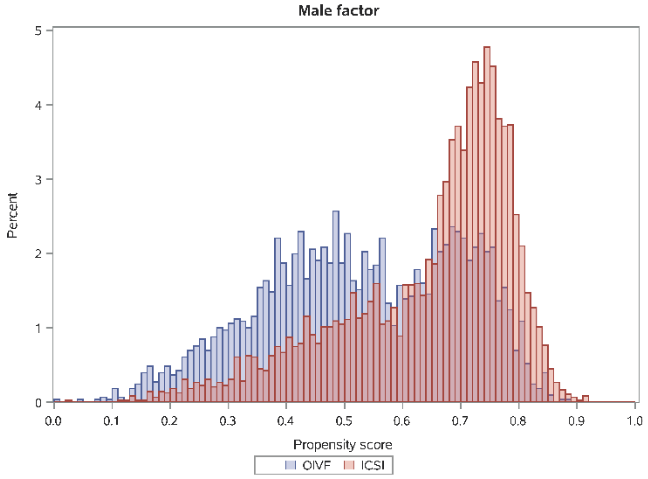

(C) Frozen-thawed vs. fresh embryo transfer

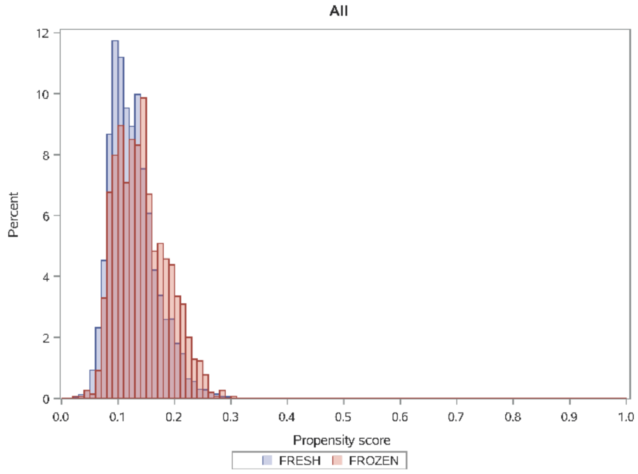

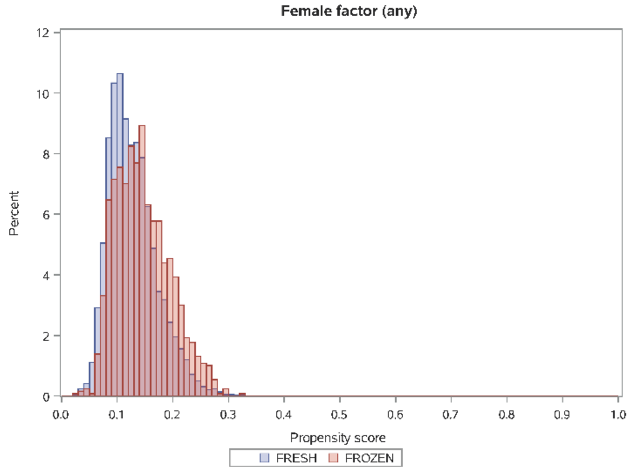

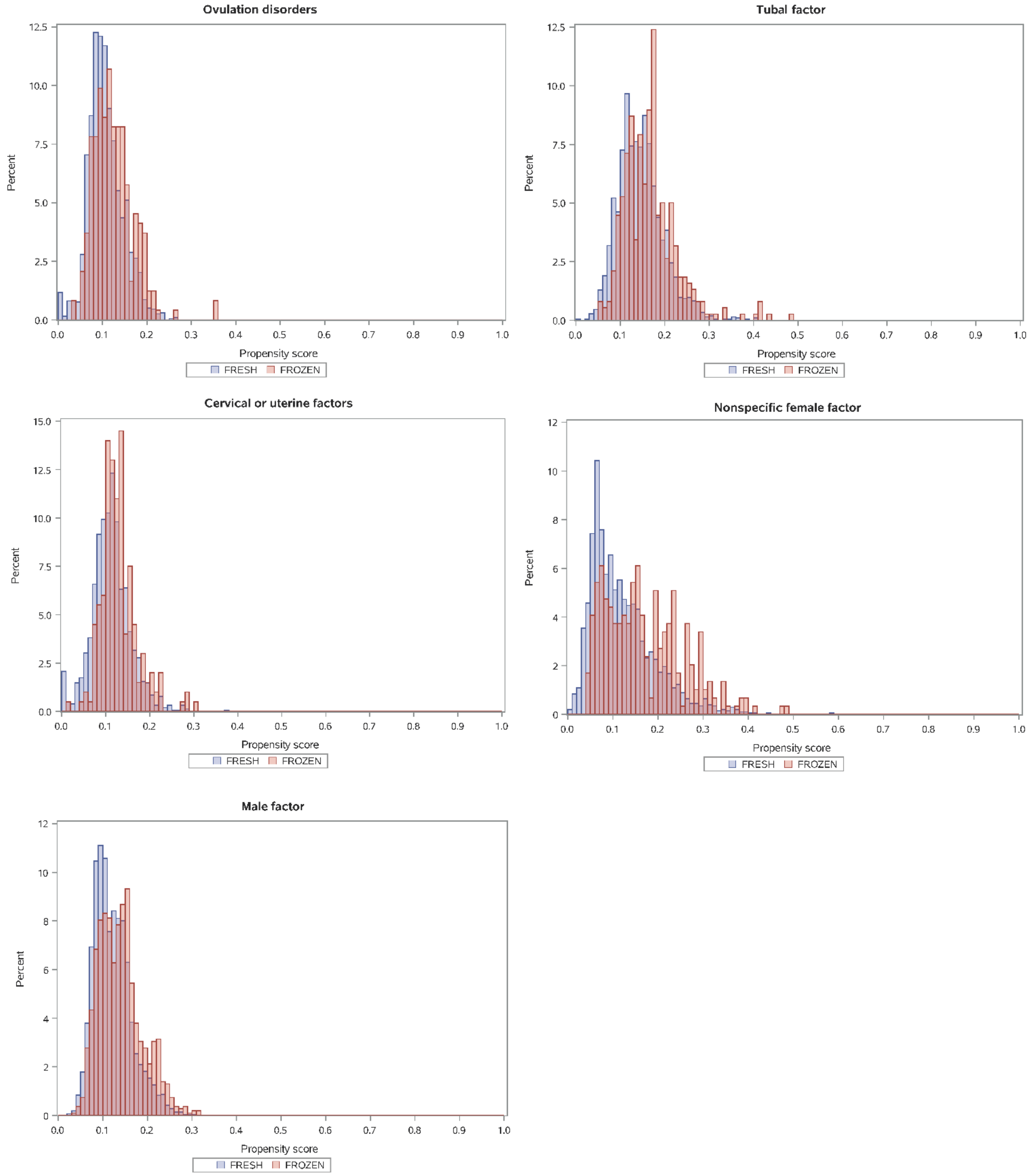
Ovulation disorders
Tubal factor
Cervical or uterine factors
Nonspecific female factor
Male factor
Percent
Propensity score
FRESH
FROZEN
0.0
0.1
0.2
0.3
0.4
0.5
0.6
0.7
0.8
0.9
1.0
2.5
5.0
7.5
10.0
12.5
15.0
0
2
4
6
8
10
12
