# Supplementary material for: Overweight or obesity in children born after assisted reproductive technologies in Denmark: A population-based cohort study
Source: PLoS Med. 2023 Dec 19;20(12):e1004324. doi: 10.1371/journal.pmed.1004324 (PMC10729995; doi:10.1371/journal.pmed.1004324)

	Crude			Adjusted			
	N of events/ N at risk	Prevalence (%)	POR (95% CI)	N of events/ N at risk	Prevalence (%)	POR (95% CI)	P-value
**All causes**							
**Overweight**							
OI/IUI	703/6478	11	Ref	659/6254	11	Ref	
Fresh	1097/10,493	10	0.93 (0.84-1.02)	1089/10,381	10	1.00 (0.90-1.10)	0.94
**Obesity**							
OI/IUI	131/6478	2.0	Ref	119/6254	1.9	Ref	
Fresh	188/10,493	1.8	0.92 (0.75-1.13)	192/10,381	1.8	0.96 (0.75-1.24)	0.78
**Female factor (any)**							
**Overweight**							
OI/IUI	584/5118	9.0	Ref	544/4925	11	Ref	
Fresh	900/8607	10	0.86 (0.77-0.95)	900/8515	11	0.96 (0.85-1.07)	0.45
**Obesity**							
OI/IUI	104/5118	2.0	Ref	94/4925	1.9	Ref	
Fresh	162/8607	1.9	0.96 (0.77-1.20)	168/8515	2.0	1.02 (0.77-1.36)	0.86
**Ovulation disorders**							
**Overweight**							
OI/IUI	210/1574	13	Ref	205/1542	13	Ref	
Fresh	207/1958	11	0.78 (0.64-0.95)	202/1950	10	0.79 (0.65-0.97)	0.02
**Obesity**							
OI/IUI	46/1574	2.9	Ref	43/1542	2.8	Ref	
Fresh	45/1958	2.3	0.90 (0.61-1.33)	47/1950	2.4	0.86 (0.55-1.34)	0.51
**Tubal factor**							
**Overweight**							
OI/IUI	39/283	14	Ref	38/267	14	Ref	
Fresh	269/2156	12	0.86 (0.63-1.20)	271/2157	13	0.89 (0.62-1.27)	0.52
**Obesity**							
OI/IUI	6/283	2.1	Ref	7/267	2.6	Ref	
Fresh	50/2156	2.3	1.39 (0.60-3.19)	50/2157	2.3	0.94 (0.38-2.33)	0.90
**Nonspecific female factor**							
**Overweight**							
OI/IUI	92/859	11	Ref	79/834	9.5	Ref	
Fresh	189/1975	9.6	0.87 (0.69-1.09)	198/1987	10	1.05 (0.81-1.35)	0.72
**Obesity**							
OI/IUI	21/859	2.4	Ref	17/834	2.0	Ref	
Fresh	24/1975	1.2	0.56 (0.33-0.94)	25/1987	1.3	0.63 (0.34-1.16)	0.14
**Male factor (any)**							
**Overweight**							
OI/IUI	332/3152	11	Ref	304/2977	10	Ref	
Fresh	804/7608	11	0.99 (0.87-1.13)	821/7616	11	1.06 (0.93-1.20)	0.41
**Obesity**							
OI/IUI	60/3152	1.9	Ref	54/2977	1.8	Ref	
Fresh	135/7608	1.8	0.98 (0.73-1.30)	143/7616	1.9	1.02 (0.74-1.41)	0.88

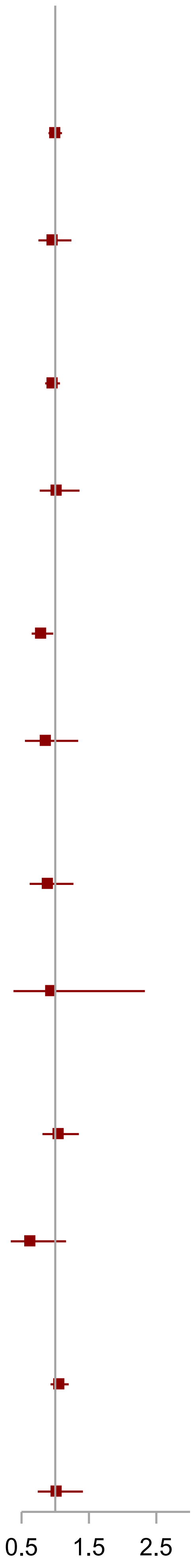

Supplement: S2 Fig — Notes: We adjusted for parental causes of infertility, maternal and paternal age at conception, maternal and paternal highest educational level at conception, maternal country of origin, maternal BMI, maternal smoking status, maternal and paternal hyperlipidemia/use of lipid-modifying drugs, maternal and paternal hypertension/use of antihypertensive drugs, diabetes (type I or II) diagnosed at any time before conception, parity, and year of conception. P values were calculated by the large-sample Wald (Z) test. Abbreviations: BMI, body mass index; CI, confidence interval; IUI, intrauterine insemination; OI, ovulation induction; POR, prevalence odds ratio. (PDF) [file pmed.1004324.s011.pdf]
