# Supplementary figures and images for: Overweight or obesity in children born after assisted reproductive technologies in Denmark: A population-based cohort study
Source: PLoS Med. 2023 Dec 19;20(12):e1004324. doi: 10.1371/journal.pmed.1004324 (PMC10729995; doi:10.1371/journal.pmed.1004324)

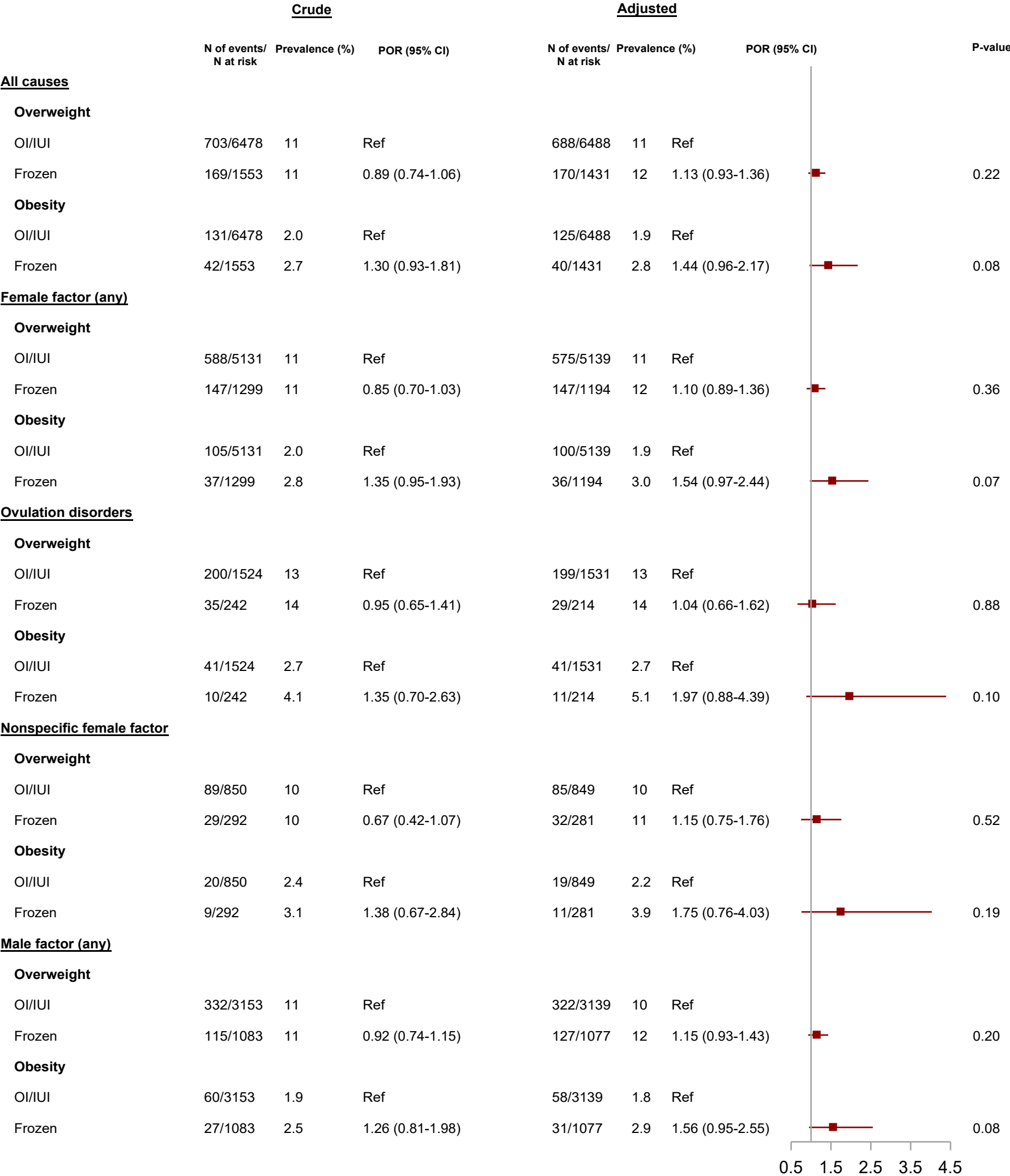

Supplement: S3 Fig — Notes: We adjusted for parental causes of infertility, maternal and paternal age at conception, maternal and paternal highest educational level at conception, maternal country of origin, maternal BMI, maternal smoking status, maternal and paternal hyperlipidemia/use of lipid-modifying drugs, maternal and paternal hypertension/use of antihypertensive drugs, diabetes (type I or II) diagnosed at any time before conception, parity, and year of conception. All standardized differences were ≤0.15 after weighting, except for maternal BMI, which yielded an standardized difference of 0.29. P values were calculated using the large-sample Wald (Z) test. Abbreviations: BMI, body mass index; CI, confidence interval; IUI, intrauterine insemination; OI, ovulation induction; POR, prevalence odds ratio. (PDF) [file pmed.1004324.s012.pdf]
