# Supplementary material for: Overweight or obesity in children born after assisted reproductive technologies in Denmark: A population-based cohort study
Source: PLoS Med. 2023 Dec 19;20(12):e1004324. doi: 10.1371/journal.pmed.1004324 (PMC10729995; doi:10.1371/journal.pmed.1004324)

A

	Crude	Adjusted
All causes	-0.05 (-0.08- -0.02)	-0.02 (-0.06-0.01); p = 0.21
Female factor (any)	-0.06 (-0.10- -0.03)	-0.03 (-0.07-0.01); p = 0.13
Ovulation disorders	-0.07 (-0.14- -0.00)	-0.08 (-0.16-0.01); p = 0.04
Tubal factor	-0.08 (-0.20-0.04)	-0.07 (-0.22-0.07); p = 0.32
Cervical or uterine factor	-0.21 (-0.32- -0.10)	-0.10 (-0.23-0.02); p = 0.11
Nonspecific female factor	-0.03 (-0.07-0.01)	0.00 (-0.09-0.09); p = 0.99
Male factor	-0.03 (-0.07-0.01)	0.01 (-0.04-0.05); p = 0.81

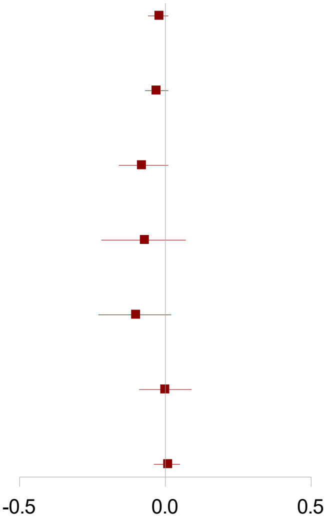

B

All causes	0.02 (-0.04-0.07)	0.04 (-0.03-0.11); p = 0.24
Female factor (any)	0.01 (-0.05-0.07)	0.04 (-0.04-0.12); p = 0.39
Ovulation disorders	0.03 (-0.12-0.18)	0.04 (-0.16-0.24); p = 0.71
Tubal factor	0.11 (-0.05-0.26)	0.11 (-0.06-0.28); p = 0.21
Cervical or uterine factor	-0.09 (-0.25-0.07)	-0.08 (-0.27-0.11); p = 0.41
Nonspecific female factor	-0.07 (-0.21-0.07)	0.09 (-0.07-0.26); p = 0.26
Male factor	0.00 (-0.07-0.07)	0.05 (-0.03-0.13); p = 0.19

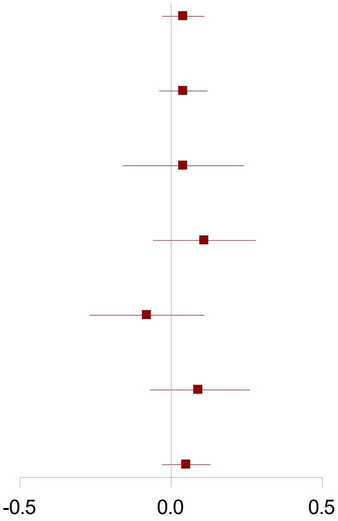

Supplement: S4 Fig — For children born after: (A) fresh embryo transfer compared to OI with or without IUI (OI/IUI) and (B) frozen-thawed embryo transfer compared to OI/IUI. Notes: We adjusted for parental causes of infertility, maternal and paternal age at conception, maternal and paternal highest educational level at conception, maternal country of origin, maternal BMI, maternal smoking status, maternal and paternal hyperlipidemia/use of lipid-modifying drugs, maternal and paternal hypertension/use of antihypertensive drugs, diabetes (type I or II) diagnosed at any time before conception, parity, and year of conception. All standardized differences were ≤0.15 after weighting, except for maternal BMI, which yielded an standardized difference of 0.29. P values were calculated by the large-sample Wald (Z) test. Abbreviation: BMI, body mass index; CI, confidence interval; IUI, intrauterine insemination; OI, ovulation induction. (PDF) [file pmed.1004324.s013.pdf]
