# Supplementary material for: Overweight or obesity in children born after assisted reproductive technologies in Denmark: A population-based cohort study
Source: PLoS Med. 2023 Dec 19;20(12):e1004324. doi: 10.1371/journal.pmed.1004324 (PMC10729995; doi:10.1371/journal.pmed.1004324)
Supplement: Attachment — Submitted filename: Response letter_final.pdf [file pmed.1004324.s014.pdf]

Dear Dr. Gaynor-Brook,

Thank you very much for your consideration of our revised manuscript. We appreciate the Reviewers' positive feedback and thoughtful comments. We believe the paper has improved following our consideration and integration of their perspectives.

Sincerely,

Kristina Laugesen on behalf of all authors

General comments from authors

Besides integrating suggestions from the Reviewers, we have made two minor changes to the analyses. Our findings and conclusion are the same as in the previous version of the manuscript. We have updated the exact estimates throughout the manuscript. We apologize for the inconvenience.

First, we have refined the definition of type 2 diabetes (covariate). In the previous version of the manuscript, we defined type 2 diabetes as a diagnostic record (from in- or outpatient hospital care) or use of antidiabetic medication (including metformin) prior to conception. However, besides type 2 diabetes, metformin is used to treat polycystic ovarian syndrome (PCOS), making the formerly applied algorithm less suitable for our study conducted in women with infertility. We believe that women with PCOS treated with metformin without a record of type 2 diabetes or use of other antidiabetic medication are more likely to receive metformin due to their PCOS rather than due to type 2 diabetes. Therefore, if women with PCOS fulfilled the prior definition of type 2 diabetes by prescriptions for metformin only (i.e. had no other record to indicate type 2 diabetes), we recoded them to not having type 2 diabetes. Consequently, the number of women with type 2 diabetes in our cohort declined (Please see Table 2), but the update had no further impact on our results.

Second, we have truncated extreme stabilized inverse probability of treatment weights at the 1st and 99th percentiles respectively, as suggested by Desai and Franklin in "Alternative approaches for confounding adjustment in observational studies using weighting based on the propensity score: a primer for practitioners" (BMJ 2019). This update had no impact on our results.

Third, we have removed the presentation of standardized differences before and after IPTW from the supplementary results, as Danish legislation does not allow us to present so-called micro data. For your convenience, we have included all updated standardized differences in the end of our response letter, showing all below 0.15. In the manuscript, we highlight that we defined balance as standardized differences ≤0.15 (Please see page 15, lines 315-316).

<u>**Comments from Editors**</u>

Comment #1

Please add "in Denmark" or similar to the title.

We have modified the title.

"Overweight or obesity in children born after assisted reproductive technologies in Denmark: A population-based cohort study". Please see page 1, line 2.

Comment #2

In the abstract, please add a new final sentence to the 'Methods and findings' subsection, which should begin "Study limitations include ..." or similar and should quote 2-3 of the study's main limitations.

We have added the following to the abstract:

"Study limitations include potential residual confounding, restriction to live births, and lack of detailed technical information about the IVF procedures." Please see page 4, lines 65-67.

Comment #3

Where available, please quote p values alongside 95% CI.

We have added p-values to the manuscript.

Comment #4

After the abstract, please add a new and accessible three-subsection 'Author summary' section in non-identical prose. You may find it helpful to consult one or two recently published research papers in PLOS Medicine to get a sense of the preferred style.

We have added an author summary to the manuscript. Please see pages 5-6.

Why was this study done?

- *The number of children born after in vitro fertilization is increasing.*
- *Information about long-term health of these children is limited and the association with Body Mass Index of the children remains controversial.*
- *Various procedures, such as type of fertilization and embryo transfer, and underlying causes of infertility may influence associations.*

What did the researchers do and find?

- *The researchers used Danish national registries to conduct a cohort study on the association between different fertility treatments and Body Mass index in children at age 5 – 8 years, taking into account underlying causes of infertility.*
- *Among children born after fertility treatment, those born after in vitro fertilization had similar Body Mass Index as those born after ovulation induction with or without intrauterine insemination.*
- *Children born after frozen embryo transfer had higher Body Mass Index and higher prevalence of obesity compared with those born after fresh embryo transfer and with those born after ovulation induction with or without intrauterine insemination. This finding was consistent across subgroups defined by underlying cause of infertility.*

What do these findings mean?

- *Our study provides reassuring results for couples who seek help to conceive.*

- *Reasons behind our findings for frozen-thawed embryo transfer need further investigation.*
- *Longer follow-up and assessment of other outcomes related to metabolic health is needed.*

Comment #5

Please state in the Methods section (Main text) whether or not the study had a protocol or pre-specified analysis plan, and if so attach the document(s) as a supplementary file, referred to in the text.

We have added the original analysis plan (S1 Text). Some changes were implemented post-hoc, including the use of inverse probability of treatment weighting, inclusion of additional potential confounders, and extra sensitivity analyses. A statement about amendments has been added to S1 Text.

Comment #6

As you have done an observational study, we ask you to avoid the word "effect" when describing your findings. For example, at line 63 that could become "no impact" or "no association between".

We have changed the wording throughout the manuscript.

Comment #7

Please ensure that the form "age 5-8 years" is used consistently throughout; likewise, "follow-up" where appropriate.

Thank you for pointing this out. We have modified the manuscript according to these suggestions.

Comment #8

While numbers should be spelt out at the start of sentences, please use numerals throughout the text (e.g., "... from 6 countries" at line 197).

Thank you. We have modified the manuscript according to the suggestion.

Comment #9

Please move the information on ethics approval from the end of the main text to the Methods section.

We have moved the information on ethics approval to the Methods section.

Comment #10

Please remove the information on competing interests, funding and data sharing from the end of the main text. In the event of publication, this information will appear in the article metadata, via entries in the submission form.

We have removed the information.

Comment #11

Throughout the text, please adapt reference call-outs to journal style: at line 85, for example, as "... past 4 decades [1,2]." (noting the absence of spaces within the square brackets).

We have adapted the reference call-outs to the journal style.

Comment #12

In the reference list, please use the journal name abbreviations "PLoS Med.", "PLoS ONE" and "BMJ".

We have used journal name abbreviations.

Comment #13

Noting reference 1, we suggest spelling out the group author name.

We have spelled out the group author name.

Comment #14

Please add full access details to reference 25.

We have added full access details to the reference.

Comment #15

Please include a completed checklist for the most appropriate reporting guideline as a supplementary file, labelled "S1_RECORD_Checklist" or similar and referred to as such in the Methods section (main text). In the checklist, please refer to individual items by section (e.g.,

"Methods") and paragraph number, not by page or line numbers as these generally change in the event of publication.

We added a completed STROBE Checklist (S1 STROBE Checklist).

<u>Comments from Reviewer #1</u>

This manuscript provides data on association of ART and adiposity in a Danish cohort

- The study cohort is unique and provides a valuable opportunity for these analyses

- Analyses are well designed and performed

- Results are important especially for public health purposes

We thank Reviewer #1 for the positive evaluation of our paper.

<u>Comments from Reviewer #2</u>

This paper investigates, in a population based registry study, if ART is associated with overweight and obesity in children born after ART in comparison to children born after non in-vitro fertilization fertility treatment (NIFT) and children born after spontaneous conception. They found that children born after ART in general had similar risk of overweight and obesity as children born after NIFT while children born after frozen embryo had a higher risk for obesity.

This is an important topic, in view of the increasing number of children born after ART worldwide and particularly after frozen embryo transfer. The ms is well written and Conclusion is careful.

We thank Reviewer #2 for the positive feedback and the careful evaluation of our study.

I have the following specific comments:

Comment #1

Children were between 5-8 years of age. How can it be that large range? Children in Denmark start (compulsory) pre-school the year they will be 6. Isn't there a more exact age when children go through these examinations? Examinations are performed at "primary school" we are told. Does that include the preschool year? Further, how is the different age of the children managed in the main analyses? Even though median age is similar.

In Denmark, the majority of children begin school (preschool class) in the calendar year when they turn 6 years of age. Children born between January and July thus begin school when they are age 6 whereas children born between August and December do so when they are age 5. This explains the lower age limit of 5. In the year before expected school start, children go through an evaluation (typically made by their pedagogues in kindergarten) and if this indicates that the child might benefit from a delayed school start (maturity issues, social skills, etc.) they are offered an extra year in kindergarten. This happens for approximately 5%-10% of Danish children, who consequentially will be 7 or 8 years of age when they begin school (depending on their month of birth). There could also be some delay from the start of school to the first routine anthropometric evaluation. This explains the higher limit of 8 years of age. Importantly, the age distributions at time of anthropometric evaluation were similar in the different exposure and comparison groups. Hence, we do not consider this age span

a major issue. Further, the International Obesity Task Force (IOTF) definitions of overweight and obesity and the World Health Organization (WHO) BMI Z-scores consider age in the calculations.

We could narrow the age span, but that would result in a higher prevalence of children with missing BMI. After considering pros and cons, we have decided to keep the original age span.

Comment #2

How about children who don't attend primary school? I presume that the 13% missing is among those attending primary school or? Reasons for children who don't attend primary school might be neurological/neurodevelopment diseases-more prevalent in ART than spontaneous conception. How large part of children do not attend primary school in the three groups? And reasons for that? Thus, can there be a selection of more "healthy" ART children in the ART group? Further, you mention that missing examination is at random. How do you know that? Might it be that obese children refuse these examination more often than normal weight children?

Thus, there are two ways of missing, those who don't attend primary school and those refusing examination. You should separate these and give us more explanations.

We apologize for the imprecise explanation in the paper. The 13% missing is among the <u>entire</u> Danish population born between 2007 and 2012 (the period of cohort establishment). The Children´s Database holds information from both health nurses and general practitioners. The percentage of children in Denmark that do not attend primary school is below 0.1% (they are home schooled) and many of these children will still attend routine checkups at their general practitioner. Therefore, non-attendance in primary school is not the explanation for the 13% with missing BMI.

According to the Danish Health Authority, who manages the database, there are three possible explanations for missing BMI: 1) the main reason is technical issues, which include randomly occurring local breakdowns of the reporting system; 2) next is that implausible reported values of height or weight are deleted as part of a routine validation process (we do not know the exact numbers deleted in this process, but the issue should be minor); 3) finally, as proposed by the Reviewer, children might refuse to be measured. From our own clinical experience this is extremely rare (and has in fact never happened in our practice), but we are not able to investigate this in a structured manner. In any case, we see no reason to suspect that any of these missing mechanisms would depend on the exposure. Indeed, the occurrence of missing BMI measurement was similar across all exposure and comparison groups in our study.

We refer to following sections of the manuscript. Some we have adapted slightly:

Methods:

"The Danish National Children´s Database records information on all children living in Denmark since 2011 and includes variables such as height, weight, and BMI categories. At age 5 – 8 years, children enroll in primary school and attend check-ups performed by health nurses. All schools in Denmark report to the database and less than 0.1% of all Danish children do not attend primary school (they are home schooled). Moreover, children attend a routine checkup at their general practitioner at age 5, and this is also reported to the database." Please see page 10, lines 192-200.

"Lack of BMI measurement was consistently 13% among children born after ART overall, conventional IVF, ICSI, frozen-thawed embryo transfer, fresh embryo transfer, or OI/IUI, and among children from the general population born after no fertility treatment. In addition, age at anthropometric evaluation was similar across groups (median age 7 years and interquartile range 6.6 – 7.6 years) (S2 Text)." Please see page 11, lines 224-229.

Discussion:

"Our study has several potential limitations. First, approximately 13% of eligible children lacked outcome measurements. Recording of weight and height in the Danish National Children´s Database comes from routine examinations of all Danish children, but missing information occurs due to technical issues, such as randomly occurring local breakdowns of reporting systems, or removal of implausible measurements as part of a central validation process. Importantly, we found that the occurrence of missing BMI measurement was similar across all exposure and comparison groups."
Please see page 29, lines 576-584.

Comment #3

I question to say that you investigate "the effect of ART".... (line 179). This is an observational study where causality cannot be established. The word "association" fits better.

We have changed the wording from effect to association throughout the manuscript.

Comment #4

I don't think you compare ART with non IVF fertility treatment. NIFT includes only ovulation induction and intrauterine inseminations. There are other kind of fertility treatment and thus not included ie surgery for myomas, uterine septas, weight loss for ovulation disorders etc. There must also be patients registered with a diagnosis of infertility but conceived spontaneously. I presume these latter are in the SC group. Should be discussed and as a limitation. Better to say that you compare ART to children born after OI and IUI.

We agree and have changed the term NIFT to ovulation induction with or without intrauterine insemination (OI/IUI) throughout the manuscript.

We have added the following paragraph to the discussion:

"Further, children born after no fertility treatment could have parents with infertility. The Danish registries do not capture time to pregnancy, but 5.4% of children in this group had parents with a prior diagnosis of infertility." Please see pages 29-30, lines 597-599.

Comment #5

How are patients, receiving ART treatment and NIFT abroad ("reproductive tourism") and delivering in Denmark managed? How big part do they constitute? And people coming from abroad, receiving fertility treatment in Denmark but not delivering there. I understand they must be excluded but this should be clarified. Reproductive tourism is quite substantial in Denmark.

Thank you for pointing this out. To our knowledge, approximately 1200 couples received fertility treatment abroad in the period of cohort establishment (2007-2012). Any live births in Denmark resulting from these would be misclassified as belonging to the general population cohort of children born after no fertility treatment. We do not know the exact number of live births following treatment abroad, but it will likely be a maximum of 0.4% of the general population cohort (calculated as 1200 divided by 305,898). We have added following to the discussion:

"Danish children born following fertility treatment abroad (i.e. reproductive tourism) would be misclassified as being born after no fertility treatment." Please see page 29, lines 596-597.

As the proportion is small and we do not perform a formal comparison to the general population, we do not consider this a major source of bias.

Indeed, a substantial number of people from abroad travel to Denmark to receive fertility treatment. However, they have no impact on the study's findings (they are not included in any of the registries).

Comment #6

Nothing is written about oocyte donation and sperm donation cycles? Are these included or excluded? Should, if excluded be presented in flow chart. If included- how do you know parental characteristics of these donors? This part needs to be clarified.

We apologize for the lack of transparency. We did not exclude children born after oocyte and sperm donation. We have added the numbers to Table 2.

Unfortunately, we do not have any information about the donors. Some characteristics (i.e. diseases or traits with a genetic component) would certainly be relevant to know whereas for other baseline characteristics (e.g., socioeconomic status, age, and smoking), it may be more relevant to consider the registered parent, rather than the biological donor. Excluding children born after treatment with donor sperm or oocytes did not change our findings. After discussing pros and cons, we decided to keep the original study population.

We have added the following clarifying text to the discussion:

"Some children in the cohort were born after fertility treatment using donor oocytes or sperm. For these, the listed baseline characteristics concerns the registered parents, not the biological donor. While this recording should be appropriate for some characteristics (i.e. parental age, educational level, and smoking), it might be less appropriate for other traits or diagnoses with a genetic component." Please see page 30, lines 600-604.

Comment #7

How were covariates identified? By statistical analyses? From the literature?-please in that case give references.

We identified the potential confounders based on subject-matter knowledge and factors reported from the literature, which we have added to the manuscript (including references):

"We identified the following potential confounders from the literature:.." Please see pages 14-15, lines 297-304.

Comment #8

Multiplicity is not mentioned? I presume this cohort includes singletons and multiples-these are more frequent in ART than SC. Multiple birth children have many different characteristics compared to singletons-known and unknown. Adjustment for multiples? Or a sensitivity analysis for singletons and /or stratification? Even though the propensity score analysis partly (probably?) takes care of that.

Yes, the cohort included both singletons and multiples. The number of children born after a singleton pregnancy was already shown in Table 2, and we have now added the number of children born after a multiple pregnancy. As the Reviewer points out, the proportion of children after multiple pregnancy is higher among children born after fertility treatment compared with children born after no treatment. In the paper, we prefer not to condition on (i.e., not adjust for, restrict to or stratify by) multiplicity, as it occurs post-exposure and might reside on the causal pathway as shown below. Controlling for the variable could introduce methodological challenges, such as collider-stratification bias.

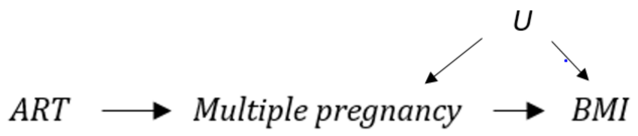

We would also like to emphasize that we only estimate causal contrasts in the cohort of children born after fertility treatment (ART vs. OI/IUI, ICSI vs. conventional IVF, and frozen vs. fresh embryo transfer) and among these the proportion born after multiple pregnancy is more similar across exposure and comparison groups. It would have been interesting to directly contrast the single vs. multiple embryo transfer interventions, but such information was not available. Nevertheless, given the Reviewer feedback, we performed a sensitivity analysis restricting to singletons, finding results very similar to the main analysis (please see below).

Table. Associations restricted to singletons.

	Adjusted POR (95% CI) for overweight	***Adjusted POR (95% CI) for obesity***	***Adjusted difference in mean BMI z-score***
ART vs. OI/IUI restricted to singletons	*1.00 (0.90-1.12)*	*0.90 (0.69-1.17)*	*-0.01 (-0.05-0.03)*
ICSI vs. conventional IVF restricted to singletons	*0.96 (0.83-1.19)*	*1.15 (0.94-1.95)*	*0.01 (-0.04-0.06)*
Frozen vs. fresh embryo transfer restricted to singletons	*1.10 (0.92-1.30)*	*1.51 (1.02-2.23)*	*0.08 (0.02-0.15)*

Comment #9

Maybe avoid words like "similar", higher etc when no statistical comparison have been made. Since I presume you regard these populations as samples. (if not no statistical comparison at all is needed). Several places in the ms.

We have modified part of the manuscript:

"Median age at follow-up was 7 years for all exposure and comparison cohorts. The crude prevalences of overweight were 10% in children born after ART, 11% in children born after OI/IUI, and 10% in children born after no fertility treatment (Fig 2). For obesity, the prevelances were 1.9%, 2.0%, and 2.7%, respectively (Fig 2). The crude prevalence of obesity depended on type of fertilization (2.0% for ICSI and 1.6% for conventional IVF) (Fig 3) and type of embryo transfer (1.8% for fresh embryo transfer and 2.7% for frozen-thawed embryo transfer) (Fig 4)." Please see pages 22-23, lines 406-416.

In the description of baseline characteristics, we have taken the liberty to preserve the original wording.

Comment #10

Table 2. Aren't there any missing for paternal BMI? I presume it is "child from a singleton pregnancy" not singleton pregnancies.

We apologize for the lack of transparency. In Table 2, we have added numbers and percentages of children with missing paternal BMI for those conceived before and after 2010 separately. This to illustrate how paternal BMI was not recorded in the Fertility Treatment Registry before 2010 (as described in the former version of the manuscript). Notably, also in children conceived from 2010 the proportion with missing paternal BMI is still quite high. Because of the missing data issue, we did not include paternal BMI as a covariate (potential confounder) in the creation of inverse probability treatment weights for our main analysis. In light of the high percentage of children with missing paternal BMI, the sixth sensitivity analysis should be evaluated with caution. We have added a footnote to the S6 Text (results from the sensitivity analyses), explaining that the percentage with missing paternal BMI was high.

We have used the term "Child from singleton pregnancy" instead.

Comment #11

In the paragraph "Overweight and obesity among children aged 5-8 born after fertility treatment and no fertility treatment" (there are no lines inserted any longer!) it is unclear when crude or adjusted results are presented. Can you clarify! In which table is this shown? Table number should be referred in text.

We apologize for the missing line numbers, which have been inserted in the revised manuscript.

In this section we describe crude prevalences of overweight and obesity in the study population. To increase transparency, we have modified this section and added figure numbers.

Section text:

"Median age at follow-up was 7 years for all exposure and comparison cohorts. The crude prevalences of overweight were 10% in children born after ART, 11% in children born after OI/IUI, and 10% in children born after no fertility treatment (Fig 2). For obesity, the crude prevelances were 1.9%, 2.0%, and 2.7%, respectively (Fig 2). In children born after ART, the crude prevalence of obesity depended on type of fertilization (2.0% for ICSI and 1.6% for conventional IVF) (Fig 3) and type of embryo transfer (1.8% for fresh embryo transfer and 2.7% for frozen-thawed embryo transfer) (Fig 4)." Please see pages 22-23, lines 402-413.

Comment #12

Suppl Table. Little strange to give Europe as ref and not Nordic? Reason? And does European mean Europe except Nordic countries?

We agree and have changed the reference to the Nordic countries in S4 Text.

Indeed, European referred to Europe except for the Nordic countries. We have added this clarification to the manuscript, Table 2 and the supplementary information.

Comment #13

Discussion

It must be wrong, in start of Discussion, sec paragraph, writing that children from no infertility treatment have a lower risk of obesity than ART. It is the other way around.

We apologize for this mistake and thank the Reviewer for noticing. We have changed the sentence:

"To provide information comparable to those of some prior studies, we also described outcomes among children born after fertility treatment and among children born after no fertility treatment, finding the first group at lower risk of obesity at age 5-8 years." Please see pages 27, lines 550-553.

Comment #14

Can you give a possible mechanism for the higher prevalence of obesity in children from frozen embryo transfer. Except speaking about "epigenetics" which myself, I am quite tired of.

We have elaborated on potential mechanisms, and only the first explanation involves epigenetics:

"Our finding of elevated obesity risk after frozen-thawed vs. fresh embryo transfer could reflect multiple pathways. First, gametes and embryos are exposed to hormones, in vitro manipulation, and culturing in artificial media during ART. Such stimuli may lead to epigenetic programming of the

metabolism of children, with a net result of increased obesity risk in a later obesogenic environment. The potential impact of epigenetic programming may differ in frozen-thawed and fresh embryos due to differences in the in vitro manipulation techniques, and hormonal and intrauterine environments. Also, the process of freezing and thawing might induce molecular changes in the embryo. Second, accumulating evidence consistently links the use of frozen-thawed embryos to larger weights at birth, which may persist into later life. Intrauterine growth conditions could be affected by various mechanisms, such as placental function, growth signalling pathways, or pregnancy complications (i.e. gestational diabetes). Third, couples with certain unknown or unmeasured traits or characteristics could be more likely to progress from fresh to frozen-thawed embryo treatment. Hence, confounding from such background factors is another possible explanation to the findings. Finally, given the multiple comparisons, our result may be a chance finding and thus needs confirmation in future studies." Please see pages 30-31, lines 610-631.

Comment #15

I don't understand you first limitation "First, its setting was within a universal tax supported healthcare system with free access to fertility treatment". But ALL IVF children are stated to be included, from publicly funded as well as private units. What is meant?

We have deleted the sentence, as it was difficult to understand. The issue is likely minor.

<u>Reviewer #3</u>

Thanks for the opportunity to review your manuscript. My role is as a statistical reviewer, so my review concentrates on the study design, data, and analysis that are presented. I have put

general questions first, followed by queries relevant to a specific section of the manuscript (with a line reference).

This manuscripts presents a large cohort study, comparing BMI (as either overweight, or obese) between children (5-8 years) born after assisted reproductive technologies (IVF and similar methods) with non-IVF technologies (ovulation induction etc.), and made comparisons between specific ART and NIFT procedures. The rationale for comparing the two categories of technology is to have a similar set of parents who have fertility issues, removing a potential source of confounding if children born after ART are compared with those born to parents who did not receive either ART or NIFT. Data comes from several linked registries that capture information on births, fertility treatment, height and weight of children, as well as inpatient visits, dispensing of medication, and demographic and civil registries. The data for the main analyses included ~327,000 children, after excluding children who did not have height and weight recorded (13% of all livebirths identified, and consistent across categories of reproductive treatment). Out of all the characteristics examined, only country of origin (with more missing/unknown for this variable in those without height/weight measurements). Data from school measurements was taken to derive BMI, which as then classified as overweight/obese based on IOTF centiles, and additionally as a continuous variable converted to a z-score (WHO). The main comparisons is children born after ART vs. NIFT, and there are secondary analyses comparing among types of ART, and fresh vs. frozen embryo transfers. A propensity score for type of reproductive therapy was estimated with logistic regression, using a wide range of relevant covariates (with information about their derivation detailed in the supplementary appendix), but not post-birth variables. The PS was then used to create IPTW weights in the substantive analysis. Standardized differences between groups in the weighted dataset were small. A range of

sensitivity analyses were considered and the rationale for these was explained. The limitations section in the discussion is appropriate for the study design and data.

I enjoyed reading this manuscript - the methodology is clear and appropriate and so is the rationale. I was amazed that the data used here is routinely collected - and a bit jealous.

We thank Reviewer #3 for spending time to review our manuscript and the positive comments.

Comment #1

After estimating the propensity score, was there evidence of common support? A figure for each analysis in the appendix that shows the distribution in scores across the relevant groups would be most effective way to demonstrate this.

Thank you for this suggestion. In the revised version of the manuscript, we have added a figure (S1 Fig) showing the distribution of propensity scores across the various exposure and comparison groups (due to its size we did not embed the figure in this response). We also provide standardized differences before and after inverse probability of treatment weighting, and believe this information fulfills the aim of showing common support.

We have modified the statistical analysis section:

"To adjust for confounding we used stabilized inverse probability of treatment weighting (IPTW) including the following steps: For each child and each comparison, we used the listed potential confounders to compute a propensity score (i.e. the predicted probability of being exposed) using a logistic regression model and then graphically displayed the distribution of propensity scores across the various exposure and comparison cohorts (S1 Fig). We then used the propensity scores to re-weight the population by stabilized IPTW and truncated the extreme weights (the 1st and 99th percentiles). This approach created a population in which the treatment assignment was independent

of the observed confounders, as these were balanced across the comparison groups. We assessed confounder balance before and after IPTW using standardized differences (SDs), with balance defined as a SD ≤0.15." Please see page 15, lines 304-316.

Comment #2

L61. This sentence was unclear - it might be better to say directly what the 'associations' is, e.g. association between different fertility treatments and overweight/obesity.

We agree and have modified the sentence:

"The associations between fertility treatments and BMI were only modestly different in the subgroups defined by the cause of infertility." Please see page 4, lines 64-65.

Comment #3

L252. Why was GEE used here? Was this to account for correlation between children born to the same parents?

We have added the following explanation to the Methods:

"Generalized estimating equations were used to account for family clustering (i.e. one woman could give birth to more than one child)." Please see pages 15-16, lines 324-324.

Comment #4

L253. I wasn't clear here why a prevalence odds ratio was estimated. I have typically seen this term used when the study design means it was not possible to distinguish between

incident and prevalent cases when the outcome is measured. With your study design, effectively all overweight/obesity is incident as the outcome is based on the first measurement recorded at school.

Because we used the available routine registration of BMI between the ages of 5 and 8, the detected cases of overweight or obesity reflect the prevalence of these conditions in this age group. I.e., it was difficult to determine if the outcome was incident or prevalent. Therefore, we have kept the term prevalence odds ratio. If the Reviewer and Editor prefer, we can change the wording.

<u>Reviewer #4</u>

General comments

This is a nationwide cohort study on BMI after different ART treatments including a cohort more than 300.000 children, whereof 113.675 were born after ART and 7728 after non-invasive fertility treatment (NIF). The authors compared the effect of different fertility treatments on BMI in children at ages 5-8 years, adjusting for and stratifying by causes of parental infertility. Crude and adjusted (using stabilized inverse probability of treatment weights) prevalences were calculated with overweight or obesity at ages 5-8 years, prevalence odds ratios (PORs) and differences in mean BMI z-scores. This paper deals with an intriguing topic and is a well conducted and well- written study however there are some concerns that need to be addressed and still needs some revision. The study comprises a large cohort of more than 300.000 children born from 2007 to 2012 including ART and children born after non-invasive fertility treatment (NIFT) and the paper adds to the accumulating knowledge on BMI after various ART procedures.

We thank Reviewer #4 for the positive comments and suggestions for improvement of the manuscript.

Comment #1

In the introduction, a short section on the risk of small-for-gestational age (SGA) and large-for-gestational-age (LGA) in children from fresh and frozen embryo transfer respectively should be added.

We agree and have modified the Introduction:

"Further, specific ART procedures might have different impacts on children. For instance, frozen-thawed embryo transfer is associated with a higher risk of being born large for gestational age, whereas fresh embryo transfer is associated with a higher risk of being born small for gestational age." *Please see page 8, lines 149-152.*

Comment #2

The authors conclude that there was no effect of ART vs. NIFT on BMI at ages 5-8 years. BMI was comparable for ICSI vs. conventional IVF. However, use of frozen-thawed embryo transfer was associated with a 1.5-fold increased risk of obesity at ages 5-8 compared to fresh embryo transfer.

The authors should make a specific analysis comparing FET vs. natural conception, as FET children are born with higher mean birth weight and are more likely to be LGA, the opposite is the case with children from fresh embryo transfer that are born with a lower mean BW and are more likely to be SGA. The BW after fresh and FET pointing in opposite directions may be translated into BMI at 5-8 years of age. Therefore, FET children should be compared with a more "neutral" control group than the children after fresh embryo transfer and this should be the group with no fertility treatment, which is also written in page 20 but this

should be emphasized more instead of the comparison with the fresh embryo transfer group. The conclusion in abstract and in the main paper should be modified accordingly.

Thank you for this comment. We agree that the direct comparison of frozen-thawed and fresh embryo transfer should be complemented with a comparison to a more "neutral group" in order to disentangle the direction of estimates. In the revised manuscript we now make sure to contrast each of the techniques with our main comparison group of children born after non-IVF fertility treatment (referred to as OI/IUI in the revised manuscript). Briefly, while those conceived without fertility treatment might be considered an even more "neutral" group, our data did not allow us to distinguish which of such births occurred to couples with infertility and which occurred to couples without infertility. In order to disentangle the potential effect of ART from the underlying infertility, we therefore identified children born after other fertility treatments (OI/IUI) as our main comparison group. As the Reviewer might have noticed, we only made descriptive/crude comparisons to the general population, as we wanted to avoid causal interpretation in the presence of confounding by indication. Further underscoring our argument, the standardized differences following IPT-weighting for this comparison were high (up to 0.8), indicating substantial confounder imbalances. In case of such imbalances, it is not appropriate to proceed with the comparison (Please see Desai and Franklin in "Alternative approaches for confounding adjustment in observational studies using weighting based on the propensity score: a primer for practitioners" BMJ 2019).

The now amended comparisons to children conceived with OI/IUI showed null associations for fresh embryo transfer and elevated risk of obesity for frozen embryo transfer (please see figures below), indicating that the previously noted excess risks with frozen compared to fresh embryo transfer is mainly driven by the frozen group (whereas children conceived with fresh embryo transfer were no different from children conceived with other fertility treatments). We have added the figures as S2 Fig, S3 Fig, and S4 Fig.

Ultimately, the choice between fresh and frozen embryo transfer should be the meaningful clinical contrast, which is why we would like to maintain this comparison in our main analysis, providing the supporting comparison to OI/IUI as a supplement. In other words, the meaningfull clinical decision is if women should be treated with fresh or frozen embryos and not (or rarely) if patients should be treated with frozen embryos or OI/IUI.

Methods:

"Additionally, we compared each type of embryo transfer to OI/IUI." Please see page 12, lines 237-238.

Results:

"The frozen-thawed vs. fresh embryo association was mainly driven by higher BMI and risk of obesity among children born after frozen-thawed embryo transfer. This was shown by comparing fresh embryo transfer with OI/IUI (adjusted POR of obesity of 0.96, 95% CI: 0.75-1.24; p = 0.78) and frozen-thawed embryo transfer with OI/IUI (adjusted POR of obesity of 1.44, 95% CI: 0.96-2.17; p = 0.08) (S2 Fig, S3 Fig, and S4 Fig), respectively." Please see page 26, lines 509-513.

Discussion:

"This nationwide population-based cohort study found no association with BMI at age 5 – 8 years when comparing ART vs. OI/IUI and ICSI vs. conventional IVF. However, use of frozen-thawed embryo transfer was associated with an excess risk of obesity compared to fresh embryo transfer and OI/IUI respectively." Please see page 27, lines 535-542.

S2 Fig. Overweight and obesity in children born after fresh embryo transfer compared to ovulation induction with or without intrauterine insemination (OI/IUI), overall and within subgroups defined by underlying causes of infertility.

	Crude			Adjusted			
	N of events/ N at risk	Prevalence (%)	POR (95% CI)	N of events/ N at risk	Prevalence (%)	POR (95% CI)	P-value
**All causes**							
**Overweight**							
OI/IUI	703/6478	11	Ref	659/6254	11	Ref	
Fresh	1097/10,493	10	0.93 (0.84-1.02)	1089/10,381	10	1.00 (0.90-1.10)	0.94
**Obesity**							
OI/IUI	131/6478	2.0	Ref	119/6254	1.9	Ref	
Fresh	188/10,493	1.8	0.92 (0.75-1.13)	192/10,381	1.8	0.96 (0.75-1.24)	0.78
**Female factor (any)**							
**Overweight**							
OI/IUI	584/5118	9.0	Ref	544/4925	11	Ref	
Fresh	900/8607	10	0.86 (0.77-0.95)	900/8515	11	0.96 (0.85-1.07)	0.45
**Obesity**							
OI/IUI	104/5118	2.0	Ref	94/4925	1.9	Ref	
Fresh	162/8607	1.9	0.96 (0.77-1.20)	168/8515	2.0	1.02 (0.77-1.36)	0.86
**Ovulation disorders**							
**Overweight**							
OI/IUI	210/1574	13	Ref	205/1542	13	Ref	
Fresh	207/1958	11	0.78 (0.64-0.95)	202/1950	10	0.79 (0.65-0.97)	0.02
**Obesity**							
OI/IUI	46/1574	2.9	Ref	43/1542	2.8	Ref	
Fresh	45/1958	2.3	0.90 (0.61-1.33)	47/1950	2.4	0.86 (0.55-1.34)	0.51
**Tubal factor**							
**Overweight**							
OI/IUI	39/283	14	Ref	38/267	14	Ref	
Fresh	269/2156	12	0.86 (0.63-1.20)	271/2157	13	0.89 (0.62-1.27)	0.52
**Obesity**							
OI/IUI	6/283	2.1	Ref	7/267	2.6	Ref	
Fresh	50/2156	2.3	1.39 (0.60-3.19)	50/2157	2.3	0.94 (0.38-2.33)	0.90
**Nonspecific female factor**							
**Overweight**							
OI/IUI	92/859	11	Ref	79/834	9.5	Ref	
Fresh	189/1975	9.6	0.87 (0.69-1.09)	198/1987	10	1.05 (0.81-1.35)	0.72
**Obesity**							
OI/IUI	21/859	2.4	Ref	17/834	2.0	Ref	
Fresh	24/1975	1.2	0.56 (0.33-0.94)	25/1987	1.3	0.63 (0.34-1.16)	0.14
**Male factor (any)**							
**Overweight**							
OI/IUI	332/3152	11	Ref	304/2977	10	Ref	
Fresh	804/7608	11	0.99 (0.87-1.13)	821/7616	11	1.06 (0.93-1.20)	0.41
**Obesity**							
OI/IUI	60/3152	1.9	Ref	54/2977	1.8	Ref	
Fresh	135/7608	1.8	0.98 (0.73-1.30)	143/7616	1.9	1.02 (0.74-1.41)	0.88

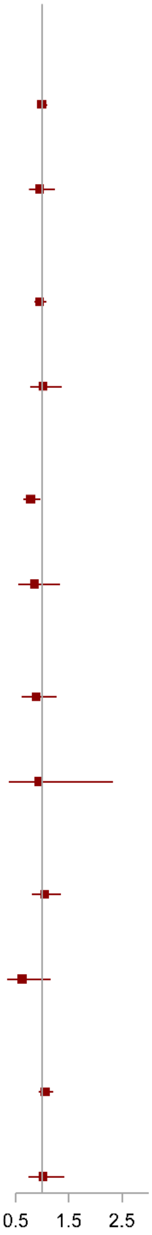

S3 Fig. Overweight and obesity in children born after frozen-thawed embryo transfer compared to ovulation induction with or without intrauterine insemination (OI/IUI), overall and within subgroups defined by underlying causes of infertility.

	Crude			Adjusted			
	N of events/ N at risk	Prevalence (%)	POR (95% CI)	N of events/ N at risk	Prevalence (%)	POR (95% CI)	P-value
**All causes**							
**Overweight**							
OI/IUI	703/6478	11	Ref	688/6488	11	Ref	
Frozen	169/1553	11	0.89 (0.74-1.06)	170/1431	12	1.13 (0.93-1.36)	0.22
**Obesity**							
OI/IUI	131/6478	2.0	Ref	125/6488	1.9	Ref	
Frozen	42/1553	2.7	1.30 (0.93-1.81)	40/1431	2.8	1.44 (0.96-2.17)	0.08
**Female factor (any)**							
**Overweight**							
OI/IUI	588/5131	11	Ref	575/5139	11	Ref	
Frozen	147/1299	11	0.85 (0.70-1.03)	147/1194	12	1.10 (0.89-1.36)	0.36
**Obesity**							
OI/IUI	105/5131	2.0	Ref	100/5139	1.9	Ref	
Frozen	37/1299	2.8	1.35 (0.95-1.93)	36/1194	3.0	1.54 (0.97-2.44)	0.07
**Ovulation disorders**							
**Overweight**							
OI/IUI	200/1524	13	Ref	199/1531	13	Ref	
Frozen	35/242	14	0.95 (0.65-1.41)	29/214	14	1.04 (0.66-1.62)	0.88
**Obesity**							
OI/IUI	41/1524	2.7	Ref	41/1531	2.7	Ref	
Frozen	10/242	4.1	1.35 (0.70-2.63)	11/214	5.1	1.97 (0.88-4.39)	0.10
**Nonspecific female factor**							
**Overweight**							
OI/IUI	89/850	10	Ref	85/849	10	Ref	
Frozen	29/292	10	0.67 (0.42-1.07)	32/281	11	1.15 (0.75-1.76)	0.52
**Obesity**							
OI/IUI	20/850	2.4	Ref	19/849	2.2	Ref	
Frozen	9/292	3.1	1.38 (0.67-2.84)	11/281	3.9	1.75 (0.76-4.03)	0.19
**Male factor (any)**							
**Overweight**							
OI/IUI	332/3153	11	Ref	322/3139	10	Ref	
Frozen	115/1083	11	0.92 (0.74-1.15)	127/1077	12	1.15 (0.93-1.43)	0.20
**Obesity**							
OI/IUI	60/3153	1.9	Ref	58/3139	1.8	Ref	
Frozen	27/1083	2.5	1.26 (0.81-1.98)	31/1077	2.9	1.56 (0.95-2.55)	0.08

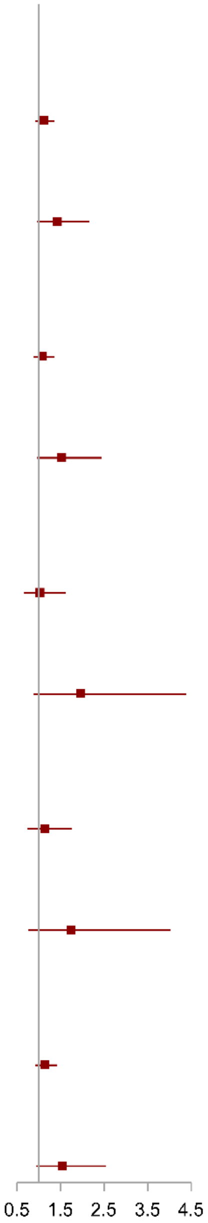

Comment #3

Regarding Table I, it is not obvious that out of 2,485 with ovulatory 58.9% were treated with ART. This needs to be specified.

We have elaborated on this in the text. Please note that 4221 children were born to women with ovulation disorder. Out of these children, 2485 (58.9%) were born after ART.

"Among the 327,301 children in our study cohort, 21,403 (6.5%) were born by parents with infertility using OI/IUI (n=7,728) or ART (n=13,675) (Fig 1). Causes of infertility varied among parents: ovulation disorders (n= 4,221 children of which 58.9% were born after ART); tubal factors (n= 3,222, 89.3% born after ART); cervical or uterine factors (n=2,557, 79.7% born after ART); male factors (n=13,097, 73.6% born after ART), and idiopathic causes of infertility (n=789, 26.1% born after ART). The type of fertility treatment differed by cause of infertility (Table 1)." Please see page 17, lines 351-357.

Comment #4

A comparison of LGA in the FET vs. the general population should be performed.

We have performed a comparison with the OI/IUI group (for rationale please see the response to comment #2) and the results are shown in the table below. However we are hesitant to include this additional outcome in our study. As the Reviewer points out (comment #1), there is already plenty of evidence on the association with birth weight. Further, we believe that adding an additional outcome to the manuscript may have a negative impact on the readability. We already have a large number of exposures, outcomes, and subgroup analyses and including one more outcome will not only add to the complexity of the manuscript but also the number of tests performed.

Table. The association between type of embryo transfer and large for gestational age (LGA).

	Crude POR (95% CI)	***Adjusted POR (95% CI)***
Frozen-thawed vs. fresh embryo transfer	*1.19 (1.04-1.36)*	*1.61 (1.36-1.90)*
Frozen-thawed embryo transfer vs. OI/IUI	*1.43 (1.23-1.67)*	*1.44 (1.16-1.79)*
Fresh embryo transfer vs. OI/IUI	*0.82 (0.74-0.91)*	*0.90 (0.78-1.04)*

Adjusted for parental causes of infertility, maternal and paternal age at conception, maternal and paternal highest educational level at conception, maternal country of origin, maternal BMI, maternal smoking status, maternal and paternal hyperlipidemia/use of lipid-modifying drugs, maternal and paternal hypertension/use of antihypertensive drugs, diabetes (type I or II) diagnosed at any time before conception, parity, and year of conception. All standardized differences were ≤0.15 after weighting, except for maternal Body Mass Index, which yielded a standardized difference of 0.29.

Comment #5

For all sub-analyses with FET, these should also be performed comparing with children from the general population.

We agree the subgroup analysis of FET should be complemented, and have performed additional comparisons shown below comment #2.

Comment #6

Discussion: It is not correct to highlight that the use of frozen-thawed embryo transfer was associated with a 1.5-fold increased risk of obesity at ages 5-8 compared to fresh embryo transfer without mentioning the comparisons to the general population.

We agree (please see our answer to comment #2) and have modified the discussion:

"This nationwide population-based cohort study found no association with BMI at age 5 – 8 years when comparing ART vs. OI/IUI and ICSI vs. conventional IVF. However, use of frozen-thawed embryo transfer was associated with an excess risk of obesity compared to fresh embryo transfer and OI/IUI respectively." Please see page 27, lines 535-542.

Comment #7

In the discussion a section on the different treatment modalities in fresh vs. frozen should be included, further a discussion on the programmed cycle FET with sequential estradiol and progesterone (and no corpus luteum) should be mentioned showing higher rates of preeclampsia and LGA (Busnelli et al., Hum Reprod 2022 and von versen-hoynck, Hypertension 2019).

Thank you, we agree and have added following to the discussion (including the references):

"Former studies reported different obstetric and perinatal outcomes depending on the specific IVF protocols. As example, Busnelli et al. and Versen-Höynck et al. found higher risk of hypertensive disorders of pregnancy, preeclampsia, post-partum haemorrhage, and caesarean section when comparing frozen-thawed embryo transfer in programmed vs. natural cycle. In our study, we found similar associations for both types of endometrial preparation." Please see page 28, lines 564-569.

Comment #8

If the authors were able to access data on medication in the IVF registry and was able to pinpoint the various endometrial preparation protocols in FET cycles that would be very interesting. These data are available.

We agree. In Denmark, two types of endometrial preparation cycles were used for frozen embryo transfer in the period 2007-2012 (period of cohort establishment): a modified natural cycle (hCG trigger only) and a programmed cycle (estrogen and progesterone). We performed a sensitivity analysis showing similar results for the two types of protocols:

Methods:

"Fifth, we separately assessed frozen-thawed embryo transfer in modified natural cycle (using hCG trigger only) and programmed cycle (estrogen and progesterone)." Please see page 16, lines 337-339.

Results:

"Frozen-thawed embryo transfer in modified natural cycle (using hCG trigger only) and programmed cycle (estrogen and progesterone) yielded similar results (S6 Text)." Please see pages 26-27, lines 531-533.

Discussion:

"Former studies reported different obstetric and perinatal outcomes depending on the specific IVF protocols. As example, Busnelli et al. and Versen-Höynck et al. found higher risk of hypertensive disorders of pregnancy, preeclampsia, post-partum haemorrhage, and caesarean section when comparing frozen-thawed embryo transfer in programmed vs. natural cycle. In our study, we found similar associations for both types of endometrial preparation." Please see page 28, lines 564-569.

Standardized differences (SDs) for assisted reproductive technologies (ART) *vs.* ovulation induction with or without intrauterine insemination (OI/IUI) before and after inverse probability of treatment weighting (IPTW), stratified by cause of infertility.

	All causes of infertility		Female factor (any)		Ovulation disorders		Tubal factor		Cervical or uterine factor		Nonspecific female factor		Male factor		Idiopathic	
	IPTW		IPTW		IPTW		IPTW		IPTW		IPTW		IPTW		IPTW	
	No	Yes	No	Yes	No	Yes	No	Yes	No	Yes	No	Yes	No	Yes	No	Yes
Year of conception																
2006-2009	-0.09	0.01	-0.08	0.01	-0.07	-0.00	-0.15	0.05	-0.06	0.03	-0.02	-0.01	-0.06	0.01	-0.12	0.04
2010-2012	0.09	-0.01	0.08	-0.01	0.07	0.00	0.15	-0.05	0.06	-0.03	0.02	0.01	0.06	-0.01	0.12	-0.04
Parity																
0	-0.00	-0.02	0.04	-0.03	0.02	-0.04	-0.07	-0.02	-0.04	-0.02	0.01	-0.00	-0.04	-0.01	0.22	0.05
≥1	0.01	-0.00	-0.03	0.03	-0.01	0.02	0.09	-0.01	0.05	0.02	-0.01	0.01	0.05	0.01	-0.23	-0.05
Cause of infertility																
Tubal factor	-0.51	0.08	-0.57	0.08	-0.42	0.03	NA	NA	-0.54	0.06	-0.16	-0.01	-0.43	0.01	NA	NA
Ovulation disorders	0.14	0.00	0.19	0.00	NA	NA	0.11	0.03	-0.02	-0.02	-0.06	0.04	0.09	0.00	NA	NA
Cervical or uterine factors	-0.27	0.08	0.29	-0.08	0.28	-0.00	-0.18	0.06	NA	NA	-0.15	-0.05	-0.20	0.04	NA	NA
Nonspecific female factor	-0.16	-0.01	-0.17	-0.02	-0.28	0.00	-0.13	-0.13	-0.14	-0.05	NA	NA	-0.12	-0.02	NA	NA
Male factor (any)	-0.49	0.04	-0.46	0.04	-0.49	0.03	-0.37	0.01	-0.26	-0.00	-0.33	-0.01	NA	NA	NA	NA
Maternal characteristics																
Age at conception (years), median	-0.33	0.00	-0.41	0.00	-0.47	0.05	-0.24	-0.01	-0.21	-0.05	-0.40	0.02	-0.22	-0.01	-0.50	-0.01
Highest educational level																
Low	0.02	-0.00	0.00	-0.01	0.04	-0.03	-0.05	0.04	0.08	0.04	0.11	-0.01	0.03	0.01	0.11	-0.10
Medium	-0.05	0.01	-0.04	0.01	-0.01	0.02	-0.06	0.04	-0.09	-0.01	0.01	0.00	-0.03	-0.01	0.02	0.05
High	0.04	-0.01	0.03	-0.01	-0.01	-0.00	0.09	-0.07	0.05	-0.01	-0.07	-0.00	0.01	0.00	-0.08	0.00
Country of origin																
Nordic	-0.00	-0.01	-0.00	-0.01	-0.01	0.01	-0.05	-0.00	-0.04	-0.04	-0.05	-0.00	-0.01	-0.01	0.00	-0.00
European (except Nordic countries)	0.00	-0.01	-0.00	-0.01	-0.01	-0.01	0.01	-0.05	0.09	-0.04	-0.00	0.07	-0.01	-0.01	-0.02	-0.06
Non-European	0.00	0.02	0.00	0.02	0.02	-0.00	0.07	0.04	-0.06	0.11	-0.00	-0.00	0.01	0.02	0.05	0.06
BMI (kg/m^2), median	0.16	0.02	0.18	0.01	0.10	0.01	0.17	0.05	0.17	-0.02	0.36	-0.02	0.18	0.02	0.06	-0.07
Smoking during pregnancy																

No	0.02	-0.03	0.03	-0.04	-0.04	-0.01	-0.03	0.00	-0.01	-0.04	0.00	-0.01	0.02	-0.02	-0.03	-0.03
Yes	-0.01	0.01	-0.01	0.01	0.05	0.01	0.01	-0.03	0.01	0.00	0.06	0.00	-0.01	0.00	0.08	0.02
Diabetes (type I, II)	0.13	0.00	0.13	0.00	0.12	-0.01	0.15	-0.03	0.08	-0.03	0.02	0.01	0.05	0.00	-0.06	-0.05
Hyperlipidaemia/ Lipid-modifying drugs	0.01	0.01	0.00	0.01	0.09	-0.03	0.10	-0.01	-0.02	0.10	0.01	0.05	0.01	0.00	0.04	-0.13
Hypertension/ antihypertensive drugs	0.04	0.00	0.03	0.00	0.02	0.00	0.06	0.01	0.06	-0.02	-0.06	-0.02	0.07	-0.00	-0.06	-0.01
Paternal characteristics																
Age at conception, median	-0.28	-0.03	-0.32	0.04	-0.30	0.07	-0.07	0.06	-0.06	-0.05	-0.36	-0.02	-0.18	0.01	-0.63	-0.06
Highest educational level																
Low	0.01	0.02	0.01	0.02	0.02	0.01	-0.01	0.01	0.05	0.02	0.06	0.02	0.04	0.01	0.01	0.06
Medium	-0.05	-0.01	-0.05	-0.01	0.00	0.00	-0.02	-0.02	-0.17	-0.02	-0.03	0.01	-0.05	-0.01	0.11	-0.06
High	0.05	-0.00	0.05	-0.01	-0.01	-0.01	0.03	0.01	0.14	0.01	-0.00	-0.03	0.02	0.00	-0.12	0.02
Hyperlipidaemia/ Lipid-modifying drugs	-0-01	0.03	-0.02	0.04	-0.02	0.02	0.15	0.01	0.00	0.00	0.01	0.05	-0.00	0.03	-0.00	0.03
Hypertension/ antihypertensive drugs	-0.02	0.01	-0.02	-0.00	0.02	0.00	-0.05	-0.05	-0.02	-0.03	-0.06	0.02	-0.03	-0.00	-0.08	0.05

Standardized differences (SDs) for frozen-thawed vs. fresh embryo transfer before and after inverse probability of treatment weighting (IPTW), stratified by cause of infertility.

	All causes of infertility		Female factor (any)		Ovulation disorders		Tubal factor		Cervical or uterine factor		Nonspecific female factor		Male factor	
	IPTW		IPTW		IPTW		IPTW		IPTW		IPTW		IPTW	
	No	Yes	No	Yes	No	Yes	No	Yes	No	Yes	No	Yes	No	Yes
Year of conception														
2006-2009	0.16	-0.01	0.17	-0.01	0.10	-0.03	0.12	0.02	0.07	-0.01	0.37	-0.03	0.20	-0.01
2010-2012	-0.16	0.01	-0.17	0.01	-0.10	0.03	-0.12	-0.02	-0.07	0.01	-0.37	0.03	-0.20	0.01
Parity														
0	0.22	-0.01	0.23	-0.01	0.20	-0.01	0.12	-0.01	0.08	0.00	0.30	-0.05	0.22	-0.02
≥1	-0.24	0.02	-0.25	0.01	-0.21	0.01	-0.14	0.00	-0.08	-0.00	-0.31	0.05	-0.24	0.02
Cause of infertility														
Tubal factor	-0.09	-0.00	-0.09	-0.00	-0.06	-0.03	NA	NA	0.02	0.03	-0.04	-0.01	-0.02	0.00
Ovulation disorders	0.08	-0.01	0.10	-0.02	NA	NA	0.07	-0.01	-0.02	-0.03	0.01	-0.09	0.07	-0.02
Cervical or uterine factors	0.05	-0.03	0.07	-0.03	-0.05	0.07	0.16	-0.01	NA	NA	0.07	-0.03	0.06	-0.03
Nonspecific female factor	0.01	0.02	0.02	0.03	-0.06	-0.01	0.01	0.04	0.07	0.05	NA	NA	0.04	0.03
Male factor (any)	0.06	0.01	0.07	0.01	0.04	-0.01	0.18	0.01	0.10	-0.02	0.13	0.04	NA	NA
Maternal characteristics														
Age at conception (years), median	-0.07	-0.01	-0.09	0.00	-0.17	0.02	-0.15	0.01	-0.01	-0.08	-0.09	-0.07	0.02	-0.02
Highest educational level														
Low	0.00	-0.01	0.00	-0.01	-0.05	0.04	0.05	0.01	-0.08	0.02	0.05	-0.05	-0.01	0.00
Medium	0.02	-0.01	0.00	-0.01	0.15	-0.04	-0.02	-0.01	-0.04	-0.05	0.04	0.02	0.04	-0.01
High	-0.02	0.01	-0.01	0.01	-0.12	0.01	-0.01	0.00	0.08	0.04	-0.07	-0.00	-0.03	0.01
Country of origin														
Nordic	0.02	0.01	0.01	0.01	0.02	0.01	-0.01	-0.01	0.02	0.01	0.00	0.02	0.01	0.02
European (except Nordic countries)	-0.02	0.00	-0.02	0.00	0.02	0.01	0.01	-0.00	-0.04	0.02	0.01	-0.03	-0.01	0.00
Non-European	-0.00	-0.02	-0.01	-0.02	-0.04	-0.01	-0.01	0.01	-0.01	0.02	0.00	-0.00	-0.01	-0.02
BMI (kg/m^2), median	0.12	-0.01	0.12	-0.02	0.10	-0.07	0.10	0.01	0.08	-0.01	0.13	0.04	0.09	-0.01
Smoking during pregnancy														
No	-0.04	0.01	-0.03	-0.00	-0.05	-0.01	-0.03	0.02	-0.10	0.05	-0.04	-0.06	-0.02	0.01
Yes	0.01	-0.00	-0.01	0.01	0.03	0.04	0.01	-0.01	0.12	-0.07	0.01	0.06	-0.01	-0.01

Diabetes (type I, II)	0.02	-0.01	0.01	-0.01	-0.02	0.02	0.06	-0.02	-0.07	-0.00	-0.10	-0.05	-0.02	-0.01
Hyperlipidaemia/ Lipid-modifying drugs	-0.03	0.01	-0.01	0.01	.	.	0.02	-0.07	0.06	0.04	-0.03	0.01	-0.05	0.01
Hypertension/ antihypertensive drugs	0.01	-0.00	0.03	0.01	-0.03	0.03	0.04	-0.02	0.02	0.01	0.12	0.04	0.02	0.01
Paternal characteristics														
Age at conception, median	-0.06	-0.00	-0.06	0.01	-0.02	-0.03	-0.09	0.02	0.05	-0.14	-0.04	-0.04	-0.01	-0.01
Highest educational level														
Low	-0.05	0.01	-0.07	0.01	-0.03	0.04	-0.08	-0.01	0.05	-0.08	-0.00	0.01	-0.06	0.01
Medium	0.04	0.00	0.06	0.01	0.13	-0.01	0.09	-0.02	0.11	0.04	-0.04	0.01	0.04	-0.00
High	-0.01	-0.01	-0.01	-0.02	-0.11	-0.01	-0.04	0.02	-0.14	0.00	0.04	-0.02	-0.00	-0.01
Hyperlipidaemia/ Lipid-modifying drugs	-0.04	0.01	-0.04	0.01	-0.02	0.01	-0.10	0.02	-0.04	0.00	0.02	-0.01	-0.03	0.01
Hypertension/ antihypertensive drugs	0.01	-0.00	-0.01	-0.00	0.03	-0.01	-0.03	0.01	-0.10	-0.09	0.03	-0.01	0.01	-0.00

Standardized differences (SDs) for intracytoplasmic sperm injection (ICSI) vs. conventional in vitro fertilization (IVF) before and after inverse probability of treatment weighting (IPTW), stratified by cause of infertility.

	All causes of infertility		Female factor (any)		Ovulation disorders		Tubal factor		Cervical or uterine factor		Nonspecific female factor		Male factor	
	IPTW		IPTW		IPTW		IPTW		IPTW		IPTW		IPTW	
	No	Yes	No	Yes	No	Yes	No	No	Yes	Yes	No	Yes	No	Yes
Year of conception														
2006-2009	0.07	-0.00	0.08	-0.00	0.05	0.02	0.11	-0.01	0.17	-0.02	0.02	-0.00	0.07	-0.01
2010-2012	-0.07	0.00	-0.08	0.00	-0.05	-0.02	-0.11	0.01	-0.17	0.02	-0.02	0.00	-0.07	0.01
Parity														
0	-0.02	-0.01	0.05	-0.01	0.05	-0.01	-0.01	-0.01	0.05	-0.04	0.21	-0.01	0.02	-0.00
≥1	0.02	0.01	-0.04	0.01	-0.02	0.00	0.00	0.01	-0.05	0.04	-0.21	0.00	-0.01	0.00
Cause of infertility														
Tubal factor	0.49	-0.03	0.41	-0.01	0.23	-0.03	NA	NA	0.27	-0.05	0.10	0.04	0.44	-0.01
Ovulation disorders	0.16	-0.03	0.03	-0.01	NA	NA	-0.05	0.01	0.01	-0.04	0.04	0.01	0.17	-0.01
Cervical or uterine factors	0.24	-0.04	0.15	-0.02	0.10	-0.04	0.01	0.00	NA	NA	-0.03	0.02	0.23	-0.02
Nonspecific female factor	0.26	-0.00	0.14	-0.00	0.12	0.00	0.00	0.03	-0.05	0.04	NA	NA	0.28	-0.00
Male factor (any)	-0.73	0.03	-0.65	0.01	-0.58	0.02	-0.60	0.01	-0.64	0.07	-0.52	0.00	NA	NA
Maternal characteristics														
Age at conception (years), median	0.21	0.01	0.13	0.01	-0.00	0.01	0.07	0.05	-0.30	0.05	0.04	0.01	0.15	-0.00
Highest educational level														
Low	0.02	-0.02	0.02	-0.01	0.01	-0.03	-0.00	0.01	0.02	0.00	0.01	-0.02	0.00	-0.00
Medium	-0.04	0.00	-0.05	0.00	-0.04	0.02	-0.05	-0.03	-0.10	-0.03	-0.04	0.00	-0.05	-0.00
High	0.03	0.01	0.04	0.01	0.04	-0.00	0.05	0.02	0.09	0.03	0.04	0.01	0.05	0.00
Country of origin														
Nordic	0.03	0.01	0.01	0.02	-0.01	0.01	0.05	0.06	0.06	0.03	-0.09	-0.00	0.06	0.01
European (except Nordic countries)	-0.01	-0.02	0.01	-0.02	0.03	-0.06	-0.06	-0.03	-0.01	-0.02	0.08	-0.01	-0.01	-0.01
Non-European	-0.03	-0.00	-0.01	-0.01	0.01	0.03	-0.01	-0.07	-0.02	-0.04	0.08	0.01	-0.07	-0.01
BMI (kg/m^2), median	-0.19	-0.03	-0.21	-0.02	-0.23	-0.01	-0.24	-0.02	-0.35	-0.02	-0.31	0.01	-0.14	-0.04
Smoking during pregnancy														
No	-0.05	0.00	-0.05	-0.00	-0.04	0.02	0.01	-0.02	0.02	-0.01	-0.04	-0.01	-0.03	-0.00

Yes	0.06	-0.01	0.07	-0.01	0.04	-0.03	0.02	0.01	-0.04	0.01	0.05	-0.00	0.06	0.01
Diabetes (type I, II)	0.04	-0.00	0.00	0.00	0.03	0.02	-0.04	0.01	-0.07	0.01	-0.05	0.00	0.03	0.00
Hyperlipidaemia/ Lipid-modifying drugs	0.02	0.01	0.00	0.01	0.00	0.01	-0.11	-0.00	0.02	0.03	0.01	0.01	-0.05	0.01
Hypertension/ antihypertensive drugs	-0.01	0.00	-0.03	0.01	-0.03	-0.00	-0.04	0.01	-0.07	0.05	-0.07	-0.00	-0.00	0.00
Paternal characteristics														
Age at conception, median	-0.08	0.03	-0.13	0.02	-0.13	0.02	-0.34	0.05	-0.43	0.04	-0.08	0.02	-0.11	0.01
Highest educational level														
Low	-0.03	0.01	-0.04	0.01	0.00	0.01	0.02	0.01	-0.06	0.04	-0.08	-0.01	-0.06	0.00
Medium	-0.07	-0.02	-0.07	-0.01	-0.06	-0.02	-0.12	-0.01	-0.02	-0.03	-0.07	-0.02	-0.06	-0.01
High	0.09	0.02	0.10	0.01	0.07	0.02	0.11	0.01	0.07	0.01	0.12	0.02	0.10	0.01
Hyperlipidaemia/ Lipid-modifying drugs	-0.05	0.01	-0.05	0.01	-0.13	0.01	-0.05	0.02	0.02	0.03	-0.02	0.01	-0.06	0.00
Hypertension/ antihypertensive drugs	-0.01	0.00	0.01	-0.00	-0.03	0.01	0.01	0.01	-0.06	0.01	-0.01	-0.01	-0.00	0.00
