# Supplementary material for: Overweight or obesity in children born after assisted reproductive technologies in Denmark: A population-based cohort study
Source: PLoS Med. 2023 Dec 19;20(12):e1004324. doi: 10.1371/journal.pmed.1004324 (PMC10729995; doi:10.1371/journal.pmed.1004324)
Supplement: Attachment — Submitted filename: Response.docx [file pmed.1004324.s015.docx]

*Dear Dr. Gaynor-Brook,*

*We are grateful for the careful evaluation of our manuscript and the opportunity to resubmit it. Below we reply to each comment, and we have revised the manuscript accordingly.*

*Sincerely,*

*Kristina Laugesen on behalf of all authors*

**Comments from Reviewer #2**

**The authors have answered most comments satisfactory and revised the manuscript accordingly.**

*We thank the Reviewer for the positive and careful evaluation of our manuscript.*

**I have a few additional comments:**

**Comment #1**

**How could restriction to live births be a limitation? Dead children cannot contribute to the follow up at 5-8 years.**

*Restriction to live births can potentially lead to the so-called live birth bias. The live birth bias is a type of selection bias (collider stratification bias) explained in more details in Liew Z, et al in “Bias from conditioning on live birth in pregnancy cohorts: an illustration based on neurodevelopment in children after prenatal exposure to organic pollutants” (Int J Epidemiol. 2015).*

*BMI can only be ascertained in live-born children. If ART affects the change of live birth and we condition on live births, we might induce a collider stratification bias in the presence of uncontrolled common causes of fetal death and BMI.*

*We have briefly elaborated on the live birth bias and added the reference.*

*“Second, our findings should be interpreted in light of restriction to live born children. If our exposures affected the probability of live birth, this restriction could lead to live birth bias in the presence of unmeasured common causes of fetal loss and high BMI. It is possible that the probability of having a live birth differed across the exposure and comparison cohorts. Because of lack of data not resulting in livebirths, we were unable to examine this issue.” Please see pages 27-28.*

**Comment #2**

**You state that 0.1% do not attend primary school in Denmark without given a reference. In Norrman et al, Human Reprod 2018 2.8% did not attend primary school. I doubt that these figures would be that different between Sweden and Denmark. Can you clarify?**

*We apologize for the lack of reference.*

*The number of children in Denmark, who are at school age, is approximately 670,000 (Statistics DK: www.statistikbanken.dk). According to a Danish report for the Danish Ministry of Education in 2018, the number of children, who are home schooled, is 475 and the number of children, who are not attending any educational offer, is 279. Unfortunately, the reference is in Danish. The percentage of 0.1% is calculated as 475 plus 279 divided by 670,000.*

*We have made a minor modification in the manuscript and added the reference:*

*“All schools in Denmark report to the database, but approximately 0.1% of all Danish children do not attend primary school (they are home schooled or do not attend any educational offer).” Please see page 10.*

**Comment #3**

**I miss a statistical comparison between ART and spontaneous conception (non infertile group). Both for ART in general and for frozen embryo transfer. I understand the authors arguments for not doing this but I still think it would strenghten the paper of several reasons. One for comparison with other studies. It can be added as a supplement and you can keep the main analysis as it is. In fact, most studies, while pinpointing the limitation, use general population as controls.**

*As suggested, we have performed the analyses as a supplement to our main analyses. The results are shown in S7 Text and S8 Text in the revised Supplementary.*

***S7 Text. Adjusted prevalences (%) and prevalence odds ratios (POR) for overweight and obesity in children born after assisted reproductive technologies (ART) vs. children from the general population.***

|  | **N at risk/**  **N of events** | **Adjusted prevalence (%)** | **Adjusted POR (95% CI)** |
| --- | --- | --- | --- |
| **ART** |  |  |  |
| **Overweight** |  |  |  |
| General population | 1355/12,279 | 11 | Ref |
| ART | 1280/12,248 | 10 | 0.95 (0.90 to 1.01); p = 0.08 |
| **Obesity** |  |  |  |
| General population | 246/12,279 | 2.0 | Ref |
| ART | 230/12,248 | 1.9 | 0.94 (0.82 to 1.07); p = 0.4 |
| **Frozen-thawed embryo transfer** |  |  |  |
| **Overweight** |  |  |  |
| General population | 163/1542 | 11 | Ref |
| Frozen-thawed embryo transfer | 7/61 | 11 | 1.03 (0.89 to 1.20); p = 0.7 |
| **Obesity** |  |  |  |
| General population | NA | NA | Ref |
| Frozen-thawed embryo transfer | NA | NA | 1.45 (1.07 to 1.96); p= 0.02 |

**Notes:** We adjusted for maternal and paternal age at conception, maternal and paternal highest educational level at conception, maternal country of origin, maternal BMI, maternal smoking status, maternal and paternal hyperlipidaemia/use of lipid-modifying drugs, maternal and paternal hypertension/use of antihypertensive drugs, diabetes (type I or II) diagnosed at any time before conception, parity, and year of conception. NA, not available due to Danish legislation concerning micro data.

***S8 Text. Adjusted differences in mean BMI Z-scores comparing children born after assisted reproductive technologies (ART) vs. children from the general population.***

|  | **Adjusted difference in mean BMI Z-score (95% CI)** |
| --- | --- |
| **ART** | -0.08 (-0.10 to -0.06); p < 0.001 |
| **Frozen-thawed embryo transfer** | 0.00 (-0.06 to 0.05); p = 0.9 |

**Notes:** We adjusted for maternal and paternal age at conception, maternal and paternal highest educational level at conception, maternal country of origin, maternal BMI, maternal smoking status, maternal and paternal hyperlipidemia/use of lipid-modifying drugs, maternal and paternal hypertension/use of antihypertensive drugs, diabetes (type I or II) diagnosed at any time before conception, parity, and year of conception.

We have added following to the text.

*We have added following to the manuscript.*

*In the section “Assisted reproductive technologies and comparison cohorts”:*

*“For context and to compare with previous literature, we also assessed associations for children born after ART vs. children from the general population born after no fertility treatment. This comparison reflects the combined effect of the treatment and the treatment indication (i.e. infertility) and not the sole effect of the treatment.” Please see page 11-12.*

*In the section “Statistical analysis”:*

*“Last, in supplemental analyses we compared children born after ART vs. children from the general population born after no fertility treatment. For these comparisons we used standardized morbidity ratio weighting instead of IPTW,* *so that estimates were the average effects of treatment and treatment indication combined among the exposed.” Please see page 16.*

*In the section “Results”:*

*“The supplemental analyses, using the general population comparator, provided similar direction of estimates as the main analyses (S7 Text and S8 Text).” Please see page 25.*

**Comment #4**

**Even though there is an increased rate of obesity in children from frozen embryo transfer, the absolute increase is rather modest. I think that should be added in Conclusion (in abstract etc)**

*Thank you for this important comment. We have modified the abstract and conclusion in the main text.*

*Abstract:*

*“Despite an elevated relative risk, the absolute risk difference was low”. Please see page 4.*

*Main text:*

*“Use of frozen-thawed embryo transfer was associated with an excess risk of obesity compared to fresh embryo transfer and OI/IUI, respectively. Despite elevated relative risks, the absolute risk differences were low.” Please see pages 29-30.*

**Comments from Reviewer #3**

**Thanks for the revised manuscript and responses my original questions. The revised manuscript covers all my original queries. I enjoyed reading this and also learning more about the level integration of health data in Denmark.**

**The adjustment to the ascertainment of DM is a good idea, I have similarly used the same strategy of only using the initiation of metformin as the start of DM when used in conjunction with other information from a study participant that occurs later.**

**I agree that putting a post-exposure variable in the analyses would not be good idea as it could create collider-stratification bias.**

*Thank you.*

**Comments from Reviewer #4**

**The authors have responded thoroughly to all the reviewers comments and it is ready for publication.**

*Thank you.*
